# A CRISPR-based chromosomal-separation technique for *Escherichia coli*

**DOI:** 10.1186/s12934-022-01957-4

**Published:** 2022-11-11

**Authors:** Junchang Su, Pengju Wang, Ju Li, Dongdong Zhao, Siwei Li, Feiyu Fan, Zhubo Dai, Xiaoping Liao, Zhitao Mao, Chunzhi Zhang, Changhao Bi, Xueli Zhang

**Affiliations:** 1 School of Biological Engineering, Dalian Polytechnic University, Dalian, 116034 China; 2 Tianjin Institute of Industrial Biotechnology, Chinese Academy of Sciences, Tianjin, 300308 China; 3 College of Life Science, Tianjin Normal University, Tianjin, 300382 China; 4 Key Laboratory of Systems Microbial Biotechnology, Chinese Academy of Sciences, Tianjin, 300308 China; 5 Biodesign Center, Key Laboratory of Systems Microbial Biotechnology, Tianjin Institute of Industrial Biotechnology, Chinese Academy of Sciences, Tianjin, 300308 China; 6 National Technology Innovation Center of Synthetic Biology, Tianjin, China

**Keywords:** Synthetic biology, Genome engineering, Chromosomal-separation, CRISPR-Cas9

## Abstract

**Background:**

Natural life systems can be significantly modified at the genomic scale by human intervention, demonstrating the great innovation capacity of genome engineering. Large epi-chromosomal DNA structures were established in *Escherichia coli* cells, but some of these methods were inconvenient, using heterologous systems, or relied on engineered *E. coli* strains.

**Results:**

The wild-type model bacterium *E. coli* has a single circular chromosome. In this work, a novel method was developed to split the original chromosome of wild-type *E. coli*. With this method, novel *E. coli* strains containing two chromosomes of 0.10 Mb and 4.54 Mb, and 2.28 Mb and 2.36 Mb were created respectively, designated as *E. coli*^0.10/4.54^ and *E. coli*^2.28/2.36^. The new chromosomal arrangement was proved by PCR amplification of joint regions as well as a combination of Nanopore and Illumina sequencing analysis. While *E. coli*^0.10/4.54^ was quite stable, the two chromosomes of *E. coli*^2.28/2.36^ population recombined into a new chromosome (Chr.4.64M^Mut^), via recombination. Both engineered strains grew slightly slower than the wild-type, and their cell shapes were obviously elongated.

**Conclusion:**

Finally, we successfully developed a simple CRISPR-based genome engineering technique for the construction of multi-chromosomal *E. coli* strains with no heterologous genetic parts. This technique might be applied to other prokaryotes for synthetic biology studies and applications in the future.

**Supplementary Information:**

The online version contains supplementary material available at 10.1186/s12934-022-01957-4.

## Background

Genome engineering is defined as the design and modification of genomic DNA sequences for a specific purpose. In 2010, U.S. scientist J. Craig Venter constructed circular chromosomes in *Saccharomyces cerevisiae* by assembling DNA fragments, and then installed those synthetic genomes into recipient *Mycoplasma* cells [1]. Since then, the progress of synthetic biology has reached milestones at the genomic scale, includes the creation of a bacterium with an entirely synthetic genome [1] and the construction of a designed yeast chromosome [2]. In 2019, the group of Zhongjun Qin fused the 16 natural chromosomes of the single-celled eukaryote *Saccharomyces cerevisiae* into a single functional chromosome [3]. This work further proved that natural life systems can be significantly modified at the genomic scale by human intervention, demonstrating the great innovation capacity of genome engineering.

The technological basis of genome engineering is the construction and implementation of novel genetic systems and remodeling of natural biological systems. In contrast to the single-chromosome *S. cerevisiae,* we aimed to design a simple technique to create an artificial multi-chromosomal prokaryotic organism, such as *E. coli*. There were reports that large epi-chromosomal DNA structures were established in *E. coli* cells. The group of G. Church employed the λ attB-attP recombination method to construct a large F-based BAC vector [4]. Starting from a minimized *E. coli* strain, Yoneji et al. split the 3 Mb chromosome into three 1 Mb chromosomes [5]. The first chromosome retains the original replication origin (oriC) and partitioning (par) system, the second one has an oriC and the par locus from the F plasmid, while the third one has the ori and par locus of the *Vibrio tubiashii* secondary chromosome. Although great achievements have been made in synthetic biology, some of these methods were inconvenient, and heterologous systems were employed, such as the F-based par locus and the BAC vector. Furthermore, since an engineered *E. coli* strain was used for the chromosome splitting, it remained unknown if wild-type strains could be manipulated using these methods.

In this work, starting from the wild-type *E. coli* strain MG1655, we aimed to develop a CRISPR-based genome engineering technique for the design and modification of genomes as desired. With this technique, we constructed a multi-chromosomal *E. coli* strain with no heterologous sequences.

## Results

### Design of a CRISPR-based chromosomal-separation technique

The key components maintaining an *E. coli* chromosome are the origin of replication and 10 replication terminators [6]. The bidirectional DNA replication of the *E. coli* chromosome is initiated from a single origin. Two replication forks, driven by the replication machinery, travel in opposite directions around the circular chromosome and terminate in a region opposite the origin. The DNA replication terminators, designated as Ter, each contain a consensus element with a length of 23 bp. Ter sites are recognized by Tus (terminus utilization substance) protein, forming a protein-DNA complex, which halts the passage of the replication fork in only one direction and permits passage by the other replication fork. A group of Ter sites with the same polarity are distributed in one half of the terminus region, and the other group, with the opposite polarity, is located in in the other half. Our first goal was to separate a 100 k bp DNA fragment out of the chromosome to form a second chromosome without perturbation of the Ter loci using a CRISPR-based genome engineering technique.

**Fig. 1:**
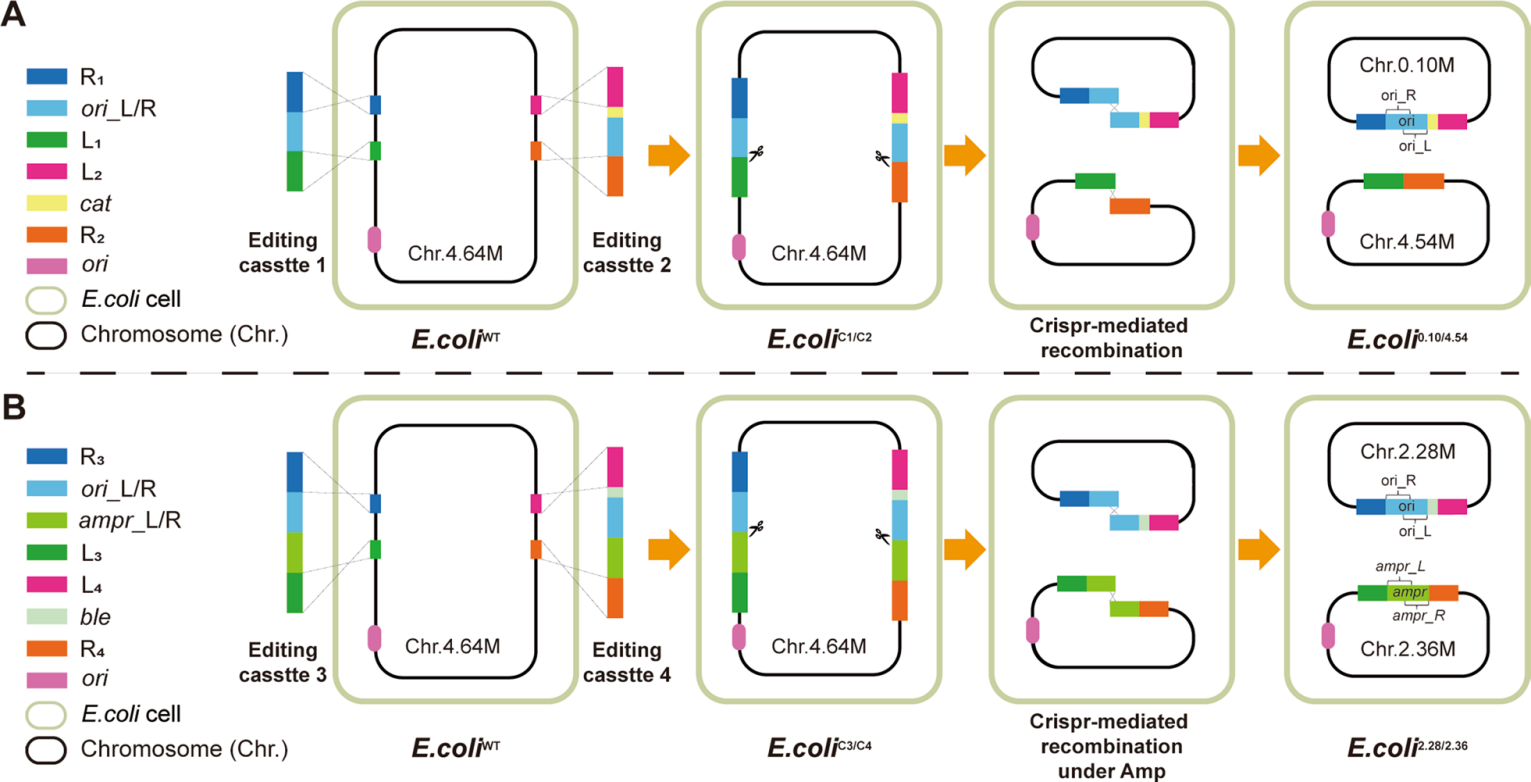
Design principle of the CRISPR-based chromosomal-separation technique developed in this study. **A** The editing cassettes 1 and 2 are inserted into the chromosome by homologous recombination, forming the *E. coli*^C1/C2^ strain. Subsequently, the CRISPR/Cas9 system is expressed to generate two double-strand breaks (DSBs) at two inserted N20PAM sequences on the editing cassettes 1 and 2, respectively. As designed, the two halves of the replication origin combine into a complete origin by recombination and form a Chr.0.10 M, while the Chr.4.54 M containing the original replication origin is constructed from the rest of the chromosome. Thus, the *E. coli*^0.10/4.54^ strain was created using this CRISPR-based chromosomal-separation technique. **B** To obtain a strain bearing two large chromosomes, the genome engineering strategy was redesigned to implement a selection pressure for cells carrying the newly created chromosomes. An ampicillin resistant gene, *amp*^*r*^*,* was separated into two halves and inserted into the chromosome along with the split *E. coli* origin of replication. Thus, after CRISPR-induced chromosomal DNA recombination, a complete *amp*^*r*^ gene was pieced together from the halves along with a second origin of replication that was also newly formed

For this purpose, we designed a CRISPR-based chromosomal-separation technique (Fig. 1). First, the two halves of the replication origin were inserted into two loci between the two groups of Ter sites on the *E. coli* chromosome. The separated second origin of replication was used to avoid the presence of two origins on a single chromosome. In the second step, two DNA double strand breaks were introduced by the Cas9/gRNA complex on the two inserted loci, which induced two intramolecular recombination events of the chromosome. As designed, the two halves of the replication origin combined into a complete origin and formed a circular DNA molecule containing all DNA sequences cleaved from the original chromosome, while the rest of the chromosome recombined into a second circular DNA molecule containing the original replication origin. Thus, two new chromosomes derived from the original one were formed in a single *E. coli* cell.

### Creation of the multi-chromosomal *E. coli*^0.10/4.54^ strain

To construct a second chromosome of 0.10 Mb, two halves of the replication origin were inserted into two loci, L_1_/R_1_ (*paa*Y, 1464 kb) and L_2_/R_2_ (*dos*P, 1564 kb), on the *E. coli* chromosome, located 0.10 Mb away from each other. After Cas9/gRNA-induced intramolecular recombination, the two halves of the replication origin recombined into a complete one, forming Chr.0.10 M containing the 0.10 Mb DNA sequence between them. At the same time, Chr.4.54 M was constructed by a recombination event between the L_1__up and L_2__low homologous arms.

**Fig. 2:**
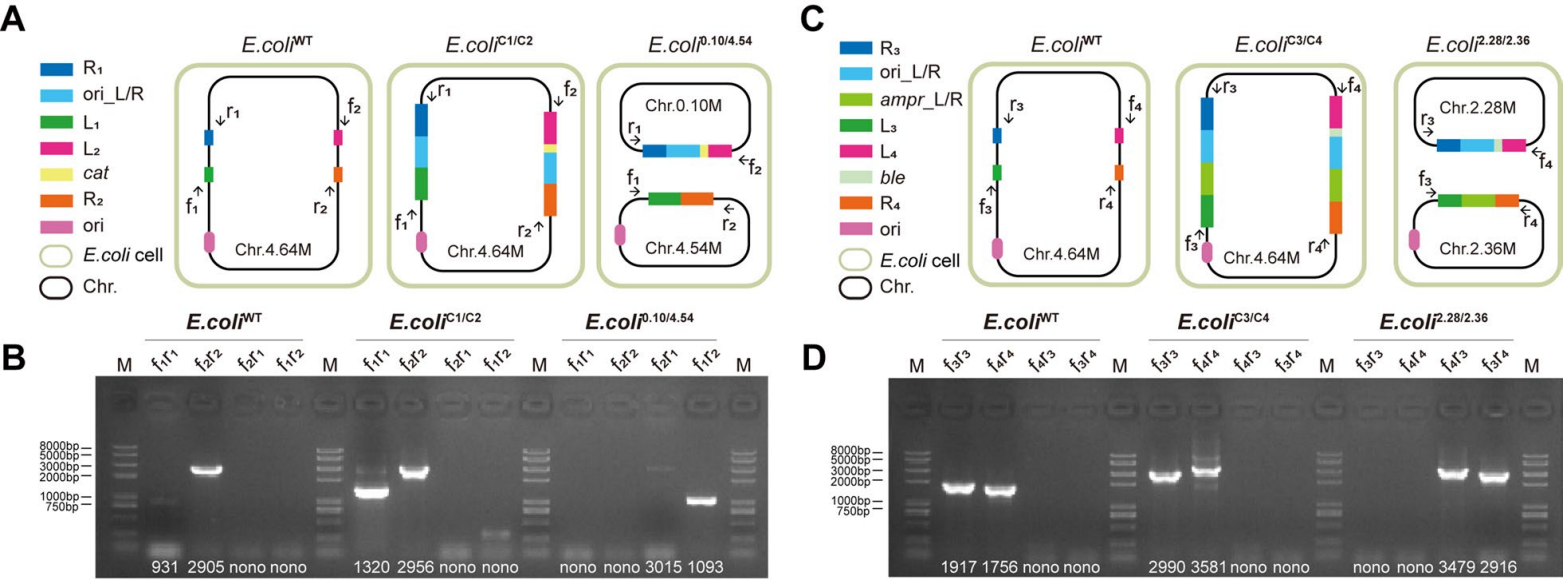
Confirmation of the genome-engineered *E. coli* stains by colony PCR. **A**, **C** Schematic showing the loci of primer pairs for colony PCR. **B**, **D** Gel pictures of colony PCR products obtained using the genomic DNA of the *E. coli* variants as template

To identify the constructed multi-chromosomal *E. coli* containing two chromosomes of 0.10 Mb and 4.54 Mb (*E. coli*^0.10/4.54^ strain), a single colony was picked for culture to ensure the purity of the population. The harvested cells were used as the template of the identification PCR. Four primer pairs were designed to amplify the key regions spanning the L_1_/R_1_, L_2_/R_2_, L_1_/R_2_ and L_2_/R_1_ sequences, respectively (Fig. 2A). In the wild-type strain *E. coli*^WT^, only the primer pairs f_1_/r_1_ and f_2_/r_2_ could generate amplification products with a size of 931 bp and 2905 bp, respectively (Fig. 2B). After integrating the editing cassettes 1 and 2, amplification products could be obtained from the *E. coli*^C1/C2^ strain using the primer pairs f_1_/r_1_ (1320 bp) and f_2_/r_2_ (2956 bp). After the CRISPR-induced recombination event, the *E. coli*^0.10/4.54^ strain was created. To confirm the successful construction, PCR was performed with another two pairs of primers as illustrated in Fig. 2A. The identification PCR was able to yield two specific PCR products with the primer pairs f_1_/r_2_ (1093 bp) and f_2_/r_1_ (3015 bp), in addition, the colony PCR bands that met the expected size were confirmed by DNA sequencing, which proved that a chromosome of 4.54 Mb and a second chromosome of 0.10 Mb were successfully split from the original chromosome of *E. coli*^C1/C2^.

**Fig. 3:**
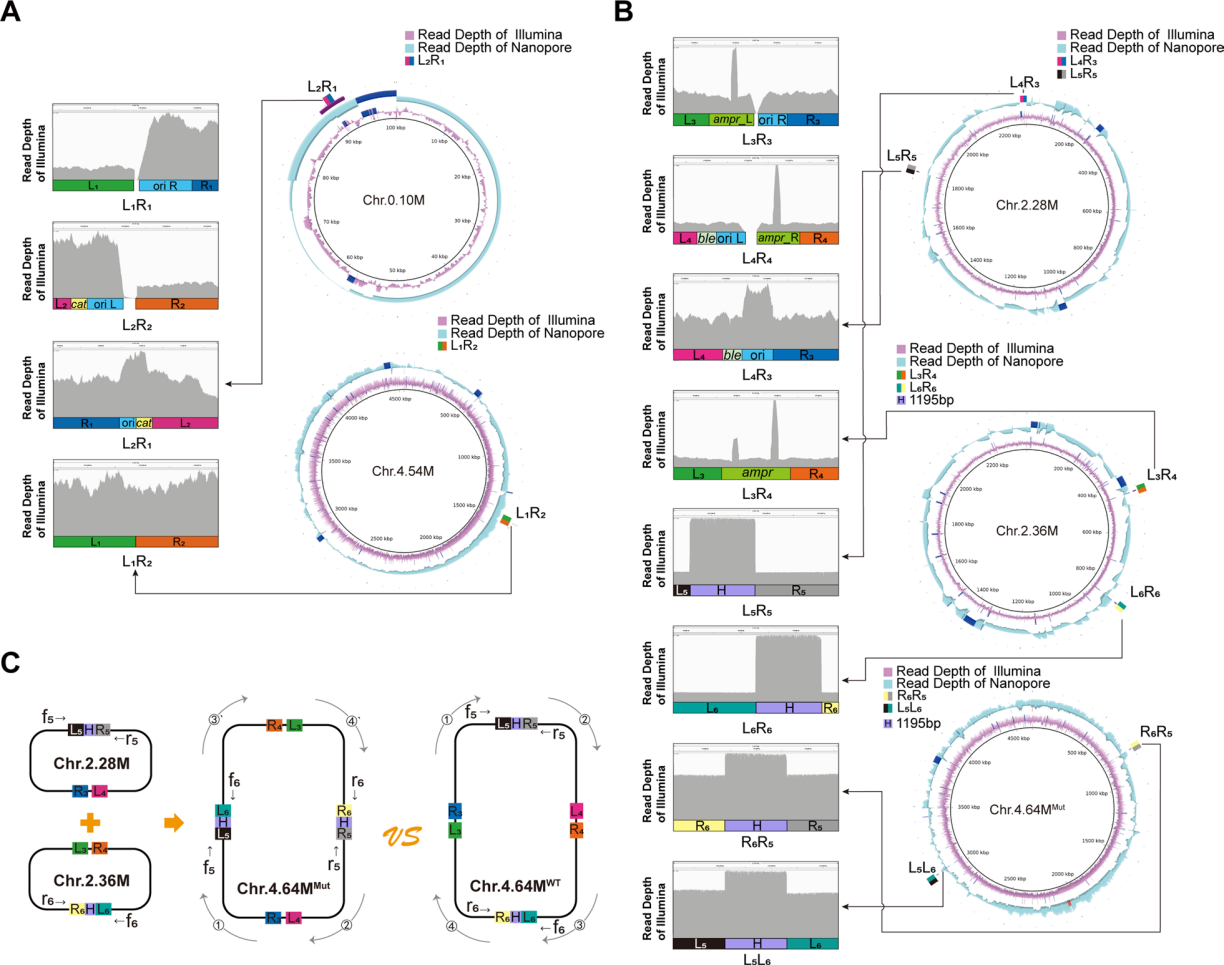
Confirmation of the genome-engineered *E. coli* stains by de novo genome sequencing. **A**, **B** Circle plots and partial enlarged views of genome-engineered *E. coli* strains. **C** The formation process in which Chr.4.64M^Mut^ was reassembled from Chr.2.28 M and Chr.2.36 M by a homologous recombination based on H, confirmed by the sequencing results, and comparison with the Chr.4.64M^WT^ genome

Large circular DNA molecules extracted in a traditional manner from cells digested in agarose plugs are often nicked/gapped and relaxed [7], and it was impossible to roughly observe the engineered chromosomal with agarose gel electrophoresis. Therefore, a combination of Nanopore sequencing (a third-generation sequencing technique) and Illumina sequencing (a second-generation sequencing technique) was carried out to further analyze the genomic DNA of the *E. coli*^0.10/4.54^ strain (Fig. 3A). The results showed that the coverage of both L_1_/R_1_ and L_2_/R_2_ regions on the genome of *E. coli*^C1/C2^ strain was broken, while the coverage of the new region L_2_/R_1_ of Chr.0.10 M and L_1_/R_2_ of Chr.4.54 M in *E. coli*^0.10/4.54^ was continuous, which proved that the CRISPR-based genome engineering technique could effectively split the *E. coli* chromosome as designed.

### Creation of the *E. coli*^2.28/2.36^ strain

Using the same strategy, we attempted to split the *E. coli* chromosome into two chromosomes with similar sizes. When PCR of the key recombinant joints was used to analyze the chromosomal configuration after genome engineering with Cas9/gRNA, we found that the designed strain containing two chromosomes was present in the culture immediately after editing. However, the newly formed chromosomes were not detected after one round of culture transfer. The result illustrated that even though *E. coli* cells with two chromosomes were created using the genome engineering technique, they were not stable, or were outcompeted by original cells during the culture process.

To obtain a strain bearing two large chromosomes, we improved the genome engineering technique by implementing a selective pressure for stains carrying the newly created chromosomes. An ampicillin resistance gene (*amp*^*r*^) was separated in two halves and inserted into the chromosome along with the split *E. coli* origin of replication. Thus, after CRISPR-induced chromosomal DNA recombination, a complete *amp*^*r*^ gene was pieced together from the halves in addition to a second origin of replication that was also newly formed (Fig. 1B). With the newly formed *amp*^*r*^ on the second chromosome, ampicillin could be added to the culture to select for cells containing both chromosomes. For identification, four primer pairs were designed to amplify the key regions spanning the newly formed L_3_/R_3_, L_4_/R_4_, L_3_/R_4_ and L_4_/R_3_ sequences, respectively (Fig. 2C). In the wild-type strain *E. coli*^WT^, only the primer pairs f_3_/r_3_ and f_4_/r_4_ could generate amplification products with a size of 1917 bp and 1756 bp, respectively (Fig. 2D). After integrating the editing cassettes 3 and 4, amplification products could be obtained from *E. coli*^C3/C4^ using the primer pairs f_3_/r_3_ (2990 bp) and f_4_/r_4_ (3581 bp).

After the recombination event, an *E. coli* strain containing two chromosomes, Chr.2.28 M and Chr.2.36 M, was created. The identification PCR was performed and the resulting PCR products were obtained with another two primer pairs as illustrated in Fig. 2D, yielding two specific PCR bands with the primer pairs f_3_r_4_ (2916 bp) and f_4_/r_3_ (3479 bp). The PCR identification experiment proved that a chromosome of 2.28 Mb and a second chromosome of 2.36 Mb were successfully split from the *E. coli*^C3/C4^ chromosome, and the resulting strain was designated as *E. coli*^2.28/2.36^.

Similarly, de novo genome sequencing using both Nanopore and Illumina methods was carried out to further analyze the genomic DNA of *E. coli*^2.28/2.36^ (Fig. 3B). As expected, Chr.2.28 M and Chr.2.36 M were successfully assembled, and the coverage of both the L_3_/R_3_ and L_4_/R_4_ regions was broken, while the coverage of the newly formed region L_4_/R_3_ of Chr.2.28 M as well as L_3_/R_4_ of Chr.2.36 M in *E. coli*^2.28/2.36^ was continuous, which proved that the CRISPR-based chromosomal-separation technique could effectively modify the *E. coli* genome as desired at the two genomic targets (*bolA* gene and *pheA* gene).

Unexpectedly, a new Chr.4.64M^Mut^ was assembled at the same time. Careful analysis revealed that Chr.4.64M^Mut^ was reassembled from Chr.2.28 M and Chr.2.36 M by a homologous recombination based on a sequence of 1195 bp (Marked as H), which was located at *ins*H-8 on Chr.2.28 M (L_5_/R_5_) and *ins*H-9 on Chr.2.36 M (L_6_/R_6_) and appeared 10 times in total on the Chr.4.64M^Mut^ chromosome (Fig. 3C). Notably, the coverage of the L_5_/R_5_ region of Chr.2.28 M, L_6_/R_6_ region of Chr.2.36 M, as well as the L_5_/L_6_ and R_6_/R_5_ regions of Chr.4.64M^Mut^ were continuous (Fig. 3B), which proved that the chromosomal organization of *E. coli*^2.28/2.36^ was unstable, with a tendency to generate Chr.4.64M^Mut^. In addition, the sequence H occurs 10 times in total across the entire *E. coli* genome, which increase the local coverage in the Illumina sequencing plots of Fig. 3B. This indicated that *E. coli* cells with two large chromosomes were not stable, which may be due to fact that *E. coli* lacks a mechanism to maintain multiple large chromosomes. This can potentially also explain why *E. coli* has a single chromosome rather than multiple chromosomes in nature. Figure 3C shows a genomic organization transition between Chr.4.64M^WT^(①-②-③-④) and Chr.4.64M^Mut^ (①-③`-④`-②).

### Stability of the engineered chromosomal organization of genome-engineered *E. coli* strains

**Fig. 4:**
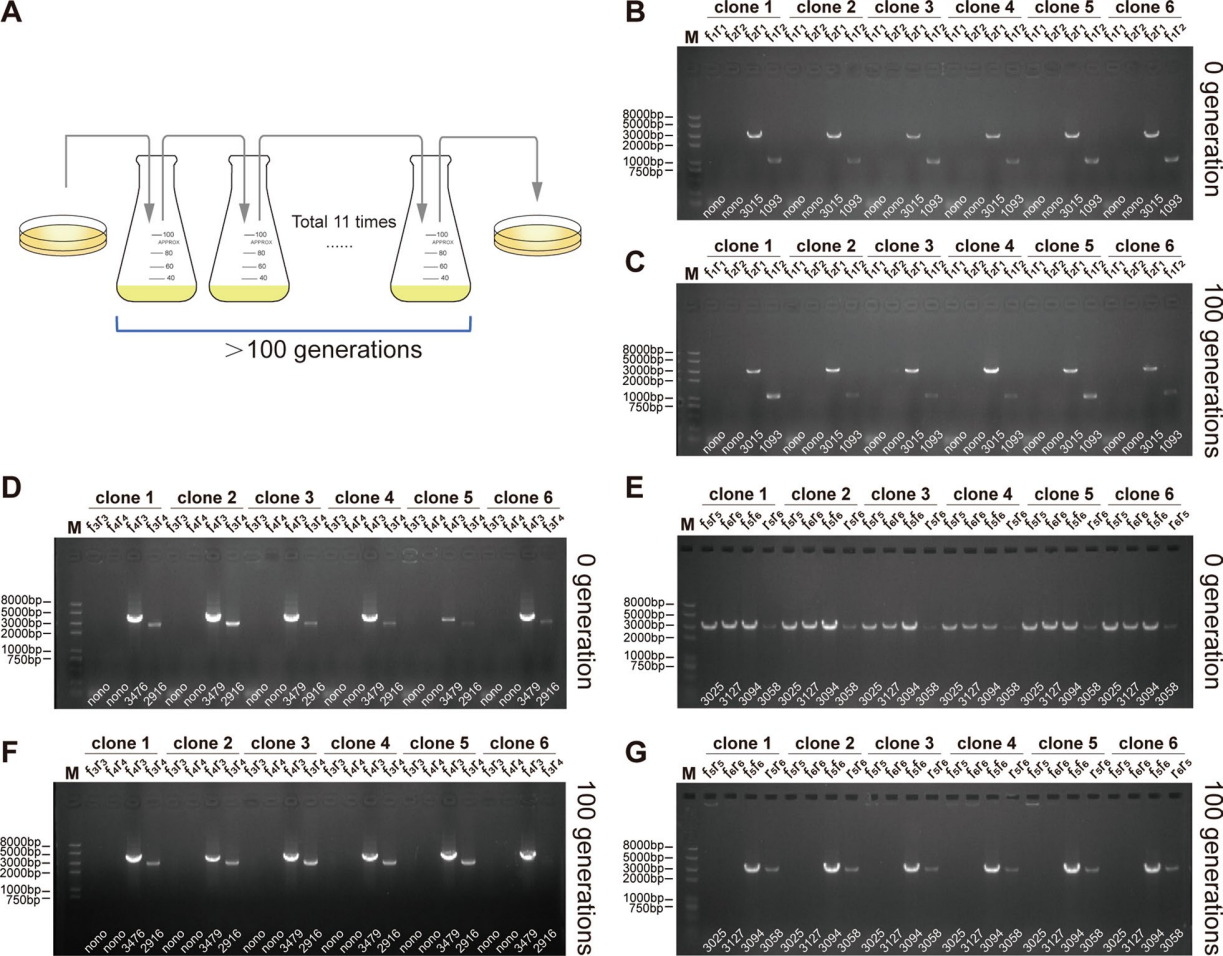
Stability of the chromosomal organization of genome-engineered *E. coli* strains. **A** Two genome-engineered *E. coli* strains were continuously transferred in batch culture at an inoculation ratio of 1‰ for 11 rounds, corresponding to approximately 100 generations. **B**, **C** Colony PCR analysis of the *E. coli*^0.10/4.54^ cells after culturing for more than 100 generations using the primer pairs f_1_/r_1_, f_2_/r_2_, f_1_/r_2_ and f_2_/r_1_. **D**, **F** Colony PCR analysis of the *E. coli*^2.28/2.36^ (mix with *E. coli*^Mut^) after culturing for more than 100 generations using the primer pairs f_3_/r_3_, f_4_/r_4_, f_3_/r_4_ and f_4_/r_3_. **E**, **G** Colony PCR analysis of the *E. coli*^2.28/2.36^ (mix with *E. coli*^Mut^) after culturing for more than 100 generations using the primer pairs f_5_/r_5_, f_6_/r_6_, f_5_/f_6_ and r_5_/r_6_

Chromosomal stability is probably the most important property of the engineered *E. coli* strain. To study the stability of the chromosomal organization, an *E. coli*^0.10/4.54^ of 0 generations that had just been identified correctly was transferred in batch culture for more than 100 generations without antibiotics (Fig. 4A), after which single colonies were subjected to PCR identification as described above to amplify the key regions spanning the L_1_/R_1_, L_2_/R_2_, L_1_/R_2_ and L_2_/R_1_ sequences, respectively. All 30 tested colonies from generations 0 and 100 maintained the *E. coli*^0.10/4.54^ chromosomal organization, leading to the conclusion that the chromosomal organization of *E. coli*^0.10/4.54^ was highly stable. The test results of 6 colonies were shown in Fig. 4B and C, the rest were shown in the Additional file 1: Fig. S1A.

Using the same method, single colonies of *E. coli*^2.28/2.36^ were subjected to PCR identification as described above to amplify the key regions spanning the L_3_/R_3_, L_4_/R_4_, L_3_/R_4_ and L_4_/R_3_ sequences, respectively. The regions modified using the genome engineering approach as designed were stable. The test results of 6 colonies were shown in Fig. 4D and F, the rest were shown in the Additional file 1: Fig. S1B.

However, the actual chromosomal organization of strain *E. coli*^2.28/2.36^ was unstable. We selected 6 single colonies of *E. coli*^2.28/2.36^ strain and used four pairs of primers to amplify the key regions spanning the L_5_/R_5_, L_6_/R_6_, L_5_/L_6_ and R_6_/R_5_ sequences. The colonies, which we subjected for colony PCR verification, were developed from the single cell containing two chromosomes, and had already grown quite a few generations. Since The genome configuration of *E. coli*^2.28/2.36^ strain was unstable, that we couldn't get a stable *E. coli*^2.28/2.36^ strain, even if the tested sample was grown from a single cell or a single colony (Fig. 4E). Further analysis showed the ratio of *E. coli*^2.28/2.36^ and *E. coli*^Mut^ was approximately 10:7 when *E. coli*^2.28/2.36^ was first created, as indicated by the sequencing coverage ratio of L_5_/R_5_: L_6_/R_6_: L_5_/L_6_: R_6_/R_5_, which was approximately 256: 249: 189: 174. After 100 generations of culture without antibiotics, Chr.2.28 M and Chr.2.36 M completely recombined back into a new chromosome, Chr.4.64M^Mut^. However, the newly formed *E. coli*^Mut^ chromosome was very stable, and no further changes were detected (Fig. 4E and G). In addition, another 24 single colonies of *E. coli*^2.28/2.36^ strain were selected to investigate the speed of transition of genomic instability with the passage of generations. Similarly, we couldn't get a stable *E. coli*^2.28/2.36^ strain from the colonies form from the 0 generation. After three batches of culture transfer for about 30 generations, 12 of the 24 single colonies completely recombined into Chr.4.64M^Mut^ via recombination; after seven batches of culture for about 70 generations, all 24 single colonies had completely recombined into Chr.4.64M^Mut^. Details are shown in Additional file 1: Fig. S2.

### Growth status of genome-engineered *E. coli* strains

**Fig. 5:**
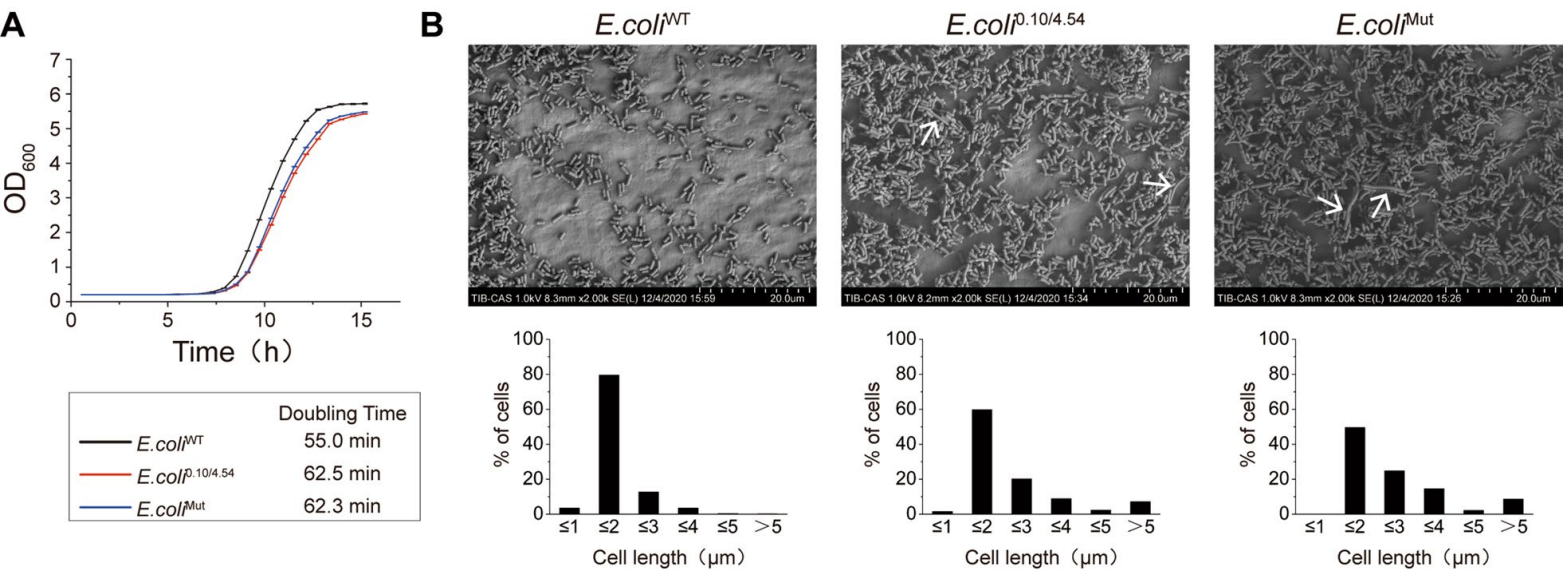
Growth status and morphological characteristics of genome-engineered *E. coli* strains. **A** Growth curves of *E. coli*^0.10/4.54^ and *E. coli*^Mut^ in 48-wells plates using LB medium, *E. coli*^WT^ was included as the positive control. Doubling times were calculated from the growth curve data. **B** Scanning electron microscopy (SEM) of *E. coli*^WT^, *E. coli*^0.10/4.54^ and *E. coli*^Mut^; The significantly elongated cells of genome-engineered *E. coli* strains were indicated by the arrows; Length distribution of the cells of *E. coli*^WT^, *E. coli*^0.10/4.54^ and *E. coli*^Mut^

Based on previous reports, the growth rate of *E. coli* and other single-cell organisms is largely limited by the speed of genome replication. To analyze the growth status of *E. coli*^0.10/4.54^ and *E. coli*^Mut^, the growth rate of the *E. coli* variants was measured during the exponential phase [8, 9]. The growth profiles of *E. coli*^WT^, *E. coli*^0.10/4.54^ and *E. coli*^Mut^ in 6 × 8 deep well plates containing LB medium without antibiotics were recorded. The growth curves were plotted as illustrated in Fig. 5A, and the corresponding doubling times were calculated. The doubling times of *E. coli*^0.10/4.54^ and *E. coli*^Mut^ in our experiment were 62.5 and 62.3 min, respectively, while the doubling time of *E. coli*^WT^ was 55.0 min. These results suggested that the *E. coli*^0.10/4.54^ and *E. coli*^Mut^ cells have a significantly slower growth rate than the *E. coli*^WT^ cells, while the doubling time of *E. coli*^0.10/4.54^ and *E. coli*^Mut^ was not significantly different.

### Morphological characteristics of genome-engineered *E. coli* strains

Since the *E. coli* chromosome was greatly changed in the multi-chromosomal and rearranged stains, we were interested to see how the changed chromosomal arrangement affected the morphological characteristics of the corresponding strains. Scanning electron microscopy was employed to study the morphological characteristics of *E. coli*^0.10/4.54^ and *E. coli*^Mut^ in the exponential phase. Compared with the WT, the cells of *E. coli*^0.10/4.54^ and *E. coli*^Mut^ were obviously elongated, while the control strain *E. coli*^WT^ exhibited the typical rod-shape (Fig. 5B). In a visual field containing a few hundred cells, the length of *E. coli*^WT^ in the exponential phase was between 2 and 3 μm, while most *E. coli*^0.10/4.54^ and *E. coli*^Mut^ cells were clearly elongated, indicated by the arrows, so that the proportions of cells with a length of 3, 4, and 5 μm were increased. Moreover, some of the engineered cells were even longer than 5 μm, suggesting that the engineered chromosome might delay the daughter-cell separation process (Fig. 5B).

## Discussion

This work provides a set of simple and rapid engineering techniques for creating multi-chromosomal *E. coli*, which might be applied to other bacterial species. While *E. coli*^0.10/4.54^ was quite stable, the two chromosomes of *E. coli*^2.28/2.36^ population recombined into a new chromosome (Chr.4.64M^Mut^), by a homologous recombination based on H sequence, which appeared 10 times in total on the Chr.4.64M^Mut^ chromosome. Deletion of all of them was not practical. Yoneji et al. obtained three 1 Mb genomes in 2021 from a very special *E. coli* strain, the 3 Mb chromosome of DGF-298 W, the *recBCD* genes and all insertion sequence (IS) of which were deleted using a stepwise genome reduction approach. DGF-298 W strain showed no auxotrophy, with fewer genes and better cell yield and better growth fitness in a rich medium than the wild type K-12 strain [10], which might perform quite differently compared with the wild-type *E. coli* MG1655 used in this study. The group of G. Church employed the λ attB-attP recombination method to construct a large F-based BAC vector [4], which were inconvenient, and heterologous systems were employed, such as the F-based par locus and the BAC vector. In this work, starting from the wild-type *E. coli* strain MG1655, we develop a CRISPR-based genome engineering technique for the design and modification of genomes as desired. With this technique, we constructed a multi-chromosomal *E. coli* strain with no heterologous sequences. The CRISPR-based chromosomal-separation technique might be further developed by combining it with other methods, such as the rapid giant DNA production in *Bacillus subtilis* [11], the multiplex genome editing in *Streptomyces* chassis [12], or the cell-free cloning system [13]. Importantly, the efficient fission of the unmodified *E. coli* genome into two defined pairs of synthetic chromosomes provides common intermediates for large-scale genome manipulations such as inversion and translocation. Precise, rapid, large-scale genome engineering operations are useful tools for creating diverse synthetic genomes.

## Conclusions

The wild-type model bacterium *E. coli* has a single circular chromosome. In this work, we developed a CRISPR-based chromosomal-separation technique to create a multi-chromosomal *E. coli*. Using this method, we first created the multi-chromosomal *E. coli*^0.10/4.54^ strain containing two chromosomes of 0.10 Mb and 4.54 Mb, respectively, which was proved by both colony PCR and a third-generation genome sequencing method. The chromosomal organization of *E. coli*^0.10/4.54^ was highly stable, and no changes were observed after more than 100 generations of culture without antibiotics. Using the same approach, the *E. coli*^2.28/2.36^ strain containing two more similarly sized chromosomes, Chr.2.28 M and Chr.2.36 M, was created. However, the chromosomal organization of *E. coli*^2.28/2.36^ was not stable, and the two large chromosomes were found to recombine into a new chromosome (Chr.4.64^Mut^) after about 70 generations of culture completely, that we couldn’t get a stable *E. coli*^2.28/2.36^ strain, even if the tested sample was grown from a single cell or a single colony. We found that both genome-engineered strains grew slightly slower than the wild type, and their cell shapes were obviously elongated to 3, 4, or 5 μm, and in some cases even longer, as observed by scanning electron microscopy.

## Methods

### Strains and culture conditions

*Escherichia coli* DH5α was used as a cloning host. Wild-type *E. coli* MG1655 was used in the genome duplication experiments. Strains were grown at 30 °C in Luria–Bertani medium (LB, 1% (w/v) tryptone, 0.5% (w/v) yeast extract, and 1% (w/v) NaCl). Kanamycin (50 mg/L), chloramphenicol (30 mg/L), ampicillin (100 mg/L), spectinomycin (100 mg/mL) and apramycin (50 mg/L) were added to the medium when appropriate. One percent (w/v) glucose and 2 g/L l-arabinose were added to the culture for the repression and induction of Cas9 expression, respectively.

*Escherichia coli* MG1655 cells with or without plasmids were grown in 50 ml of LB medium supplemented with the appropriate antibiotics at 30 °C to OD_600_ = 0.6, and then made electro competent by concentrating 100-fold and washing three times with 10% ice-cold glycerol. Then, 50 ng of plasmid DNA or 800 ng of the editing cassette was used for electroporation. The shocked cells were resuspended in 1 mL of LB, incubated for 2 h at 30 °C, and spread on LB agar plates with the appropriate antibiotics.

### Plasmid construction

The plasmids pgRNA(L_1_/R_1_) and pgRNA(L_2_/R_2_) used for the creation of the *E. coli*^0.10/4.54^ strain were constructed using Golden Gate assembly [14], for which DNA primers were designed using J5 Device Editor [15]. The inducible gRNA plasmids pgRNA(L_1_/R_1_) and pgRNA(L_2_/R_2_) were constructed for guiding CRISPR/Cas9 to two target loci, L_1_/R_1_ (*paa*Y, 1464 kb) and L_2_/R_2_ (*dos*P, 1564 kb), on the chromosome of *E. coli* MG1655. The backbones of plasmids pgRNA(L_1_/R_1_) and pgRNA(L_2_/R_2_) were PCR amplified from pACYC184-M [16], and the gRNA with its promoter was amplified from the plasmid pRed_Cas9_ΔpoxB300 [17]. The plasmid pgRNA(N20PAM) was constructed using the same method. Plasmid pRedCas9 for the inducible expression of λ-Red and Cas9 was modified from pRed_Cas9_Δ*pox*B300 and assembled using the Golden Gate method. For the creation of the *E. coli*^2.28/2.36^ strain, the inducible gRNA plasmids pgRNA(L_3_/R_3_) and pgRNA(L_4_/R_4_) were constructed using the same method for guiding CRISPR/Cas9 to two target loci, L_3_/R_3_ (*bol*A, 454 kb) and L_4_/R_4_ (*phe*A, 2738 kb), on the chromosome of *E. coli* MG1655.

### Construction of the editing cassettes

The editing cassette 1 (C1) and editing cassette 2 (C2) used for the creation of *E. coli*^0.10/4.54^ were constructed using Golden Gate assembly. Four modularized parts were prepared with optimized 4-nt linkers that can be processed using type IIS restriction enzymes for assembly of the editing cassette 1, which included a left homologous arm (L_1_) from the left part of the *paaY* gene on the chromosome, a CRISPR/Cas9 recognition region (N20PAM), the right half of the replication origin (ori_R), and a right homologous arm (R_1_) from the right part of the *paaY* gene on the chromosome. Five modularized parts were also prepared with same method for assembly of the editing cassette 2, which included a left homologous arm (L_2_) from the left part of the *dosP* gene on the genome, a complete chloramphenicol resistance gene (*cat*), the left half of the replication origin (*ori*_L), a CRISPR/Cas9 recognition region (N20PAM), and a right homologous arm (R_2_) from the right part of the *dosP* gene on the chromosome. Modularized parts of editing cassette 1 and editing cassette 2 were modified and optimized for construction of editing cassette 3 (C3) and editing cassette 4 (C4) for the creation of *E. coli*^2.28/2.36^. In the construction of editing cassette 3, *amp*^*r*^_L was added to the editing cassette 1, L_1_ was replaced with a left homologous arm (L_3_) from the left part of the *bolA* gene, and R_1_ was replaced with a right homologous arm (R_3_) from the right part of the *bolA* gene. At the same time, editing cassette 4 was constructed, in which *amp*^*r*^_R was added to the editing cassette 2, the *cat* gene was replaced with a complete bleomycin resistance gene (*ble*), L_2_ was replaced with a left homologous arm (L_4_) from the left part of the *pheA* gene, and R_2_ was replaced with a right homologous arm (R_4_) from the right part of the *pheA* gene.

There were four pairs of identical sequences of 40 bp. After CRISPR/Cas9 cleavage induced homologous recombination, one pair at the end of L_1_ and at the front of R_2_, which was used to cyclize chromosome Chr.4.54 M; two pairs at the end of *ori*_L and at the front of *ori*_R, which were used to reconstruct the entire replication origin and cyclize two chromosomes: Chr.0.10 M and Chr.2.28 M respectively; the last pairs at the end of *amp*^*r*^_L and at the front of *amp*^*r*^_R, which were used to reconstruct the entire ampicillin resistance gene and cyclize chromosome Chr.2.36 M. Four Golden Gate reactions were performed to assemble these parts into the corresponding editing cassettes. The L and R homologous arms of each editing cassette (about 500 bp each) were amplified from the genomic DNA of *E. coli* MG1655. The selection markers (*amp*^*r*^_L, *amp*^*r*^_R, *ble*, and *cat*) with the CRISPR/Cas9 recognition region (N20PAM) were PCR-amplified from the plasmids pETDuet1 pPICZαA and pACYCDuet1 with the N20PAM sequence embedded in the reverse primer.

All the DNA templates were PCR-amplified using Phusion polymerase (New England BioLabs, USA). PCR products were purified by preparative agarose gel electrophoresis using the AxyPrep DNA Gel Extraction Kit (Axygen Biosciences, USA), and the template was digested with *Dpn*I before assembly. Primers for the construction of the plasmids and editing cassettes, as well as other primers are summarized in Additional file 1: Table S2.

### Chromosome separating procedure

The genome engineering process is illustrated in Fig. 1. *Escherichia coli* MG1655 competent cells harboring pRedCas were prepared by inducing the expression of the CRISPR-Cas9 system and λ-RED proteins using l-arabinose. An aliquot comprising 50 μL of the competent cells was mixed with 50 ng of pgRNA(L_1_/R_1_) and 800 ng of editing cassette 1 DNA in a 2-mm Gene Pulser cuvette (Bio-Rad, USA). After electroporation at 2.5 kV and immediate resuspension in 1 mL of ice-cold LB medium, the cells were incubated for 2 h at 30 °C, and then spread on LB agar plates with kanamycin and apramycin. For each editing experiment, ten transformants were identified by colony PCR with the f_1_/r_1_ primer pairs illustrated in Fig. 2A, a forward primer upstream of the left homologous arm, and a reverse primer downstream of the right homologous arm. The expected PCR products were subjected to DNA sequencing for further confirmation. A correct clone was transferred into LB medium with kanamycin and grown overnight at 42 °C to eliminate the temperature-sensitive pgRNA(L_1_/R_1_) plasmid with the apramycin resistance gene. A single colony that grew on kanamycin plates but did not grow with apramycin was selected for subsequent experiments. Subsequently, the Haploid^C1/C2^ *E. coli* strain with chloramphenicol resistance was obtained by integrating editing cassette 2 into the *E. coli* chromosome in the same way as described above. Finally, the *E. coli*^C1/C2^ harboring pRedCas9 was then transformed with the pgRNA(N20PAM) plasmid through electroporation. Transformants were grown in LB medium at 30 °C with appropriate antibiotics for 2 h, after which 2 g/L l-arabinose was added to induce the expression of the CRISPR-Cas9 system and λ-RED proteins. After overnight culture, the cells were spread on LB agar plates with kanamycin, apramycin and chloramphenicol. To identify correctly edited clones, ten colonies were analyzed by colony PCR with three primer pairs designed to amplify the key regions as illustrated in Fig. 2A, and the PCR products were subjected to DNA sequencing for further confirmation. Eventually, the artificial *E. coli*^0.10/4.54^ was created. After finishing all genome modifications, all editing plasmids were eliminated by growing overnight at 42 °C.

The construction of *E. coli*^2.28/2.36^ and *E. coli*^0.10/4.54^ was slightly different. In the last step, a CRISPR/Cas9 system was expressed with gRNA(N20PAM) to induce two double-strand breaks (DSBs) at two N20PAM sequences inserted as part of the editing cassettes 3 and 4, which caused a DSBs-mediated intra-chromosomal recombination event between the *amp*^*r*^_L and *amp*^*r*^_R homologous arms. Thus, a new ampicillin resistance gene was generated in *E. coli*^2.28/2.36^ that was not present in *E. coli*^0.10/4.54^. To identity correctly edited clones, ten colonies were analyzed by colony PCR with three primer pairs designed to amplify the key regions as illustrated in Fig. 2E, and the PCR products were subjected to DNA sequencing for further confirmation. Eventually, the artificial *E. coli*^2.28/2.36^ was created. After finishing all genome modifications, all editing plasmids were eliminated by growing overnight at 42 °C.

### De novo genome sequencing of two genome-engineered *E. coli* strains

The whole genomes of the two engineering *E. coli* strains were sequenced using a Nanopore sequencing platform. Cells of *E. coli*^0.10/4.54^ and *E. coli* ^2.28/2.36^ were harvested, and all further experimental procedures were carried out according to the standard protocol provided by Oxford Nanopore Technologies (ONT), including sample quality control, library construction, library quality control and library sequencing. In the assembly process, the genome was assembled using high-accuracy Illumina data (Q30 > 85%) and nanopore reads using Unicycler (0.4.8) and Tricycler (0.5.3) software to obtain high quality contigs, and finally Illumina reads were used for error correction in Pilon software.

### Investigation of the chromosomal stability of two genome-engineered *E. coli*

Two engineered *E. coli* and wild-type *E. coli* were continuously transferred in batch culture at a 1‰ inoculation ratio without antibiotics, and cultured overnight for 16–18 h to OD_600_ = 5. A total of 11 rounds of inoculation were conducted, corresponding to approximately 100 generations. The culture of the first (0 generation), third (about 30 generations), fifth (about 50 generations), seventh (about 70 generations), ninth (about 90 generations) and last (about 100 generations) round of *E.coli*^2.28/2.36^ were spread on LB agar plates to investigate the speed of transition of genomic instability with the passage of generations, while the first and last round culture of wild-type *E. coli* and *E.coli*^0.10/4.54^ cultures were spread on LB agar plate to investigate the chromosomal stability. Thirty colonies from the each LB agar plate were examined by colony PCR, using the above mentioned sets of identification primers.

### Calculation of the exponential growth rate

Two engineered *E. coli* and wild-type *E. coli* were cultured without antibiotics. The growth rates during the exponential phase were evaluated according to the growth curves. The doubling time (DT) was calculated based on two continuous reading points in a growth curve, according to the equation, below:

$$DT=\left(tj-ti\right)/{log}_{2}\left(\frac{Cj}{Ci}\right)$$

where Ci and Cj represent the two OD_600_ values at two continuous time points of tj and ti, which were at intervals of either 0.5 or 1 (h) in the present study. Every four to five continuous growth rates that exhibited the largest mean and the smallest standard deviation were averaged to calculate the exponential doubling time for the growth curve.

### Investigation of cell morphology by scanning electron microscopy

For scanning electron microscopy (SEM), cells of the wild-type *E. coli* and two engineered *E. coli* in the exponential phase were harvested, and washed three times with phosphate buffered saline (PBS, pH = 7.2). The sample were fixed for 2 h in 2.5% glutaraldehyde and post-fixed for 1 h with 1% of osmium tetroxide. The samples were dehydrated with ethanol and dried in an Automated Critical Point Dryer (Leica EM CPD300). Then, the samples were coated with platinum and observed under a scanning microscope (Hitachi SU8010).

## Supplementary Information

**Additional file 1**: **Table S1.** Plasmids and *E. coli* stains used in this study. **Table S2.** Primers used in this study. **Table S3.** The schematic and sequences of the editing cassette used in this study. **Table S4.** The sequences of clone PCR products used to identity the *E. coli* variants. **Figure S1.** Stability of the chromosomal organization of *E. coli*^0.10/4.54^ and *E. coli*^2.28/2.36^. (A) Colony PCR analysis of the *E. coli*^0.10/4.54^ cells after culturing for more than 100 generations using the primer pairs f_1_/r_1_, f_2_/r_2_, f_1_/r_2_ and f_2_/r_1_. (B) Colony PCR analysis of *E. coli*^2.28/2.36^ after culturing for more than 100 generations using the primer pairs f_3_/r_3_, f_4_/r_4_, f_3_/r_4_ and f_4_/r_3_. **Figure S2.** Stability of the chromosomal organization of *E. coli*^2.28/2.36^ (mix with *E. coli*^Mut^). A total of 11 rounds of inoculation were conducted, corresponding to approximately 100 generations. The culture of the first (0 generation), third (about 30 generations), fifth (about 50 generations), seventh (about 70 generations), ninth (about 90 generations) and last (about 100 generations) round of *E. coli*^2.28/2.36^ (mix with *E. coli*^Mut^) was spread on LB agar plates, 24 single colonies of each strain were selected and four pairs of primers f_5_/r_5_, f_6_/r_6_, f_5_/f_6_ and r_5_/r_6_ were used to investigate the speed of transition of genomic instability with the passage of generations.

## Data Availability

We provide supporting and necessary data for publication of the article. All supporting data is present in the article and the additional documents. The strains and plasmid associated with this work will be made physically available by the authors upon reasonable request. The raw sequencing data have been submitted to the NCBI Sequence Read Archive (SRA: PRJNA793343). Correspondence and requests for materials should be addressed to C.Z, C.B. and X.Z. (zhangcz@dlpu.edu.cn; bi_ch@tib.cas.cn; zhang_xl@tib.cas.cn).

## Abbreviations

CRISPR: Clustered regular interspaced short palindromic repeat
WT: Wild-type
*E. coli*: *Escherichia coli*
Chr.: Chromosome
Mut: Mutation
Ter: The DNA replication terminators
Tus: Terminus utilization substance
DSBs: Double-strand breaks
*Amp*^*r*^: *Ampicillin* resistant gene
*Ble*: *Bleomycin* resistant gene
*Cat*: *Chloramphenicol* resistant gene
Gel: Agarose gel
PCR: Polymerase chain reaction
Ori: Origin of replication
