# Supplementary material for: A CRISPR-based chromosomal-separation technique for Escherichia coli
Source: Microb Cell Fact. 2022 Nov 11;21:235. doi: 10.1186/s12934-022-01957-4 (PMC9652834; doi:10.1186/s12934-022-01957-4)
Supplement: Supplementary file 1 — Additional file 1: Table S1. Plasmids and E. coli stains used in this study. Table S2. Primers used in this study. Table S3. The schematic and sequences of the editing cassette used in this study. Table S4. The sequences of clone PCR products used to identity the E. coli variants. Figure S1. Stability of the chromosomal organization of E. coli0.10/4.54 and E. coli2.28/2.36. (A) Colony PCR analysis of the E. coli0.10/4.54 cells after culturing for more than 100 generations using the primer pairs f1/r1, f2/r2, f1/r2 and f2/r1. (B) Colony PCR analysis of E. coli2.28/2.36 after culturing for more than 100 generations using the primer pairs f3/r3, f4/r4, f3/r4 and f4/r3. Figure S2. Stability of the chromosomal organization of E. coli2.28/2.36 (mix with E. coliMut). A total of 11 rounds of inoculation were conducted, corresponding to approximately 100 generations. The culture of the first (0 generation), third (about 30 generations), fifth (about 50 generations), seventh (about 70 generations), ninth (about 90 generations) and last (about 100 generations) round of E. coli2.28/2.36 (mix with E. coliMut) was spread on LB agar plates, 24 single colonies of each strain were selected and four pairs of primers f5/r5, f6/r6, f5/f6 and r5/r6 were used to investigate the speed of transition of genomic instability with the passage of generations. [file 12934_2022_1957_MOESM1_ESM.docx]

**Additional file 1**

**Table S1. Plasmids and *E. coli* stains used in this study**

| **Name** | **Description** | |
| --- | --- | --- |
| **Plasmids** | |  |
| pgRNA(L_1_/R_1_) | | expression of a gRNA guiding CRISPR/Cas9 to a target locus (*paaY*) on *E. coli*^WT^ genome |
| pgRNA(L_2_/R_2_) | | expression of a gRNA guiding CRISPR/Cas9 to a target locus (*dosP*) on *E. coli*^WT^ genome |
| pgRNA(L_3_/R_3_) | | expression of a gRNA guiding CRISPR/Cas9 to a target locus (*bolA*) on *E. coli* ^WT^ genome |
| pgRNA(L_4_/R_4_) | | expression of a gRNA guiding CRISPR/Cas9 to a target locus (*pheA*) on *E. coli* ^WT^ genome |
| pgRNA(N20PAM) | | expression of a gRNA guiding CRISPR/Cas9 to N20PAM regions of the editing cassettes |
| pRedCas9 | | expression of CRISPR-Cas9 system and λ-RED proteins |
| **Strains** | |  |
| *E. coli*^WT^ | | *E. coli* MG1655 (wild type stain) |
| *E. coli*^C1/C2^ | | *E. coli* MG1655 genome containing editing cassette 1 and editing cassette 2 |
| *E. coli*^0.10/4.54^ | | *E. coli* MG1655 cell containing Chr. 0.10M and Chr. 4.54M |
| *E. coli*^C3/C4^ | | *E. coli* MG1655 genome containing editing cassette 3 and editing cassette 4 |
| *E. coli*^2.28/2.36^ | | *E. coli* MG1655 cell containing Chr.2.28M & Chr.2.36M |
| *E. coli*^Mut^ | | *E. coli* MG1655 cell containing Chr.4.64^Mut^ |

**Table S2. Primers used in this study**

| **Name** | **Sequence** | **Description** |
| --- | --- | --- |
| pgRNAf_1_ | ccaggtctcatagcccagctaattacctgattgtgttttagagctag | Construction of pgRNA(L_1_/R_1_) plasmid |
| pgRNAr_1_ | ccaggtctcagctaagatctgactcc |  |
| pgRNAf_2_ | ccaggtctcatagcgctggtaaaccagtttcagtgttttagagctag | Construction of pgRNA(L_2_/R_2_) plasmid |
| pgRNAr_2_ | ccaggtctcagctaagatctgactcc |  |
| pgRNAf_3_ | ccaggtctcatagcgcgggccgttttgttttgtcgttttagagctag | Construction of pgRNA(L_3_/R_3_) plasmid |
| pgRNAr_3_ | ccaggtctcagctaagatctgactcc |  |
| pgRNAf_4_ | ccaggtctcatagctgatgtgaatcatccggcacgttttagagctag | Construction of pgRNA(L_4_/R_4_) plasmid |
| pgRNAr_4_ | ccaggtctcagctaagatctgactcc |  |
| pgRNAf_5_ | ccaggtctcatagctagtccatcgaaccgaagtagttttagagctag | Construction of pgRNA(N20PAM) plasmid |
| pgRNAr_5_ | ccaggtctcagctaagatctgactcc |  |
| L_1_f | ttatcagatagacggtctgactccggttgtgc | Construction of editing cassette 1 |
| L_1_r | ccaggtctcattcgatggactactgccgacaatcaggtaattagctgg |  |
| oriR_f | ccaggtctcacgaaccgaagtatggtatccaaggaacaacggttgttcag |  |
| oriR_r | ccaggtctcagcttttgagtctgttgacccatacg |  |
| R_1_f | ccaggtctcaaagcagggtacgcatgagt |  |
| R_1_r | gtgaattataggaaagtatgtttgattagataataa |  |
| L_2_f | ccacggctgtgattagaaccgacc | Construction of editing cassette 2 |
| L_2_r | ccaggtctcactggtacccgccgaagaaattcc |  |
| *cat*_f | ccaggtctcaccagcatgtgcagctccatcagcaaaag |  |
| *cat*_r | ccaggtctcactccttacgccccgccctgccactcatc |  |
| ori_Lf | ccaggtctcaggagatattccggacaacctttctcctttctatgaagc |  |
| ori_Lr | ccaggtctcacggttcgatggactagtgatcctggaccgtataagctgggatc |  |
| R_2_f | ccaggtctcaaccgaagtatggccaaagcagaaatgccagctaattacc |  |
| R_2_r | tgcgctatcgaaagtgagcgccgagg |  |
| L_3_f | aatcgcccctggtaaaagaaac | Construction of editing cassette 3 |
| L_3_r | ccaggtctcacggtaaatccagacaaaacaaaacggcc |  |
| *amp^r^*_Lf | ccaggtctcaaccgacgtcaggtggcacttttc |  |
| *amp^r^*_Lr | ccaggtctcagtgcatgatcccccatgttgtgcccaggtctcagcac |  |
| ori_Rf | tagtccatcgaaccgaagtatggtatccaaggaacaacggttgttc |  |
| ori_Rr | ccaggtctca gtgc ttgagtctgttgacccatacgc |  |
| R_3_f | ccaggtctcagcacgttttgttttgtctggattttgcgc |  |
| R_3_r | caaactctacagagtaagtgaagtc |  |
| L_4_f | atcaatacggtctacagcc | Construction of editing cassette 4 |
| L_4_r | ccaggtctcacggttaataatccagtgccgg |  |
| *ble*_f | ccaggtctcaaccggttgacaattaatcatcggc |  |
| *ble*_r | ccaggtctcagtgcgcaaattaaagccttcgag |  |
| ori_Lf | ccaggtctcagcacagatattccggacaacctttctcc |  |
| ori_Lr | ccaggtctcacggtccatacttcggttcgatggactagtgatcctggaccgtataagc |  |
| *amp^r^*_Rf | ccaggtctcaaccggaaggagctaaccgcttttttg |  |
| *amp^r^*_Rr | ccaggtctcagtgcgaagatcctttgatcttttctacgg |  |
| R_4_f | ccaggtctcagcaccatccggcactggattattactgg |  |
| R_4_r | gccattacaggccatgctggtgg |  |
| f_1_ | ttggcggcttgaatattgaacagg | Confirm of *E. coli*^0.10/4.54^ |
| r_1_ | taaagaaagccggatgcactaacc |  |
| f_2_ | ccaccagtcattatcgcattcag |  |
| r_2_ | tacattcgtcacaaccgtgaagg |  |
| f_3_ | ttgcagattaaccgggccattcggac | Confirm of *E. coli*^2.28/2.36^ |
| r_3_ | cgatggtgaactcttcgccagctttgtg |  |
| f_4_ | gatcgtgaacgcgatttgctggaaag |  |
| r_4_ | cgatgccggaatggtgattgttagtg |  |
| f_5_ | ctgcgccataaacctgtagcacacc | Confirm of *E. coli*^Mut^ |
| r_5_ | ctgggatacgctgaagcgttgactc |  |
| f_6_ | gatctgctcgacgttcgcgcattac |  |
| r_6_ | ctggtcaaccaacattgcccactcc |  |

**Table S3. The schematic and sequences of the editing cassette used in this study.**

**Editing cassette 1**

ttatcagatagacggtctgactccggttgtgccagaagagagttttgtccatccgacagcggtattgatcggcgatgttattctcggcaagggcgtttacgttgggccaaatgccagcctgcgtggcgattttggtcgtatcgtggtgaaagatggcgcgaacattcaggataattgcgttatgcacggttttcccgagcaggatactgttgtaggagaagatggacatattggtcatagcgctatccttcacggctgcattatccgccgcaatgcattagtgggaatgaacgcggtagtgatggacggtgcggtgattggcgagaacagcattgttggtgcatccgcatttgtgaaagccaaagcagaaatgccagctaattacctgattgtcggcagtagtccatcgaaccgaagtatggtatccaaggaacaacggttgttcagtttttgagttgtgtataacccctcattctgatcccagcttatacggtccaggatcaccgatcattcacagttaatgatcctttccaggttgttgatcttaaaagccggatccttgttatccacagggcagtgcgatcctaataagagatcacaatagaacagatctctaaataaatagatcttctttttaatacccaggatcccaggtctttctcaagccgacaaagttgagtagaatccacggcccgggcttcaatccattttcataccgcgttatgcgaggcaatcaccatgttttatccggatccttttgacgtcatcatcattggcgggggtcatgcaggcaccgaggccgcgatggccgcggcgcgtatgggtcaacagactcaaaagcagggtacgcatgagtaccaggtgctggtgacacgctgtaagcagacgttacatcaagtcgagccattgcgggaaattgaacctggcaggaaacgcctggtatttgatgagaatctgcgaccgaaacagtaacagatgtaaaattattttgtccctttaattataaagcagagttatgtttaagctctgctttatttatttgagtattaattcataccgttttttcatcatataattatttataatgagaatgtggttttaatttgtaatttatattatatacacaatttatatatttcatggtctttttttattcacctgaattataattgtgaattataggaaagtatgtttgattagataataa

**Editing cassette 2**

ccacggctgtgattagaaccgaccgtcacatcaacaacatattgcgggtctgggcaggcttgccacaacatcgcctgtgcttgttgtgggcgatacgcatcgtaaatcaccagttgtaaccctgacagctgggcgatgctgatacttttcgccagcgcggtaatcgcatccttgtgtaacagacaacgcgcttgctgataaatagctttacctgtgatgttatcagcgcaggcgtatttcaattcgatctccagatcagggaagattacggctaaatcaaccagttcggtggtatccgacataaacggttcctgtataagacaaaaattgctgcgctttccgcttatgcagatctcatgccatgccgggataagcgccagaatgctggcttaaagttatattttttaacttttgatcaacatttgtgcagcgtagtgcagttttggtgcaagaggggaagttaaggaaggaatctcccggaatcgtagctgaaatcacagtatttaagtgacagtgtcacgttaaatgaaaacccgcgagtgcgggcgagaggaatttgtcagattttcagcggtaacacgctgctcatccagcctggaatttcttcggcgggtaccagcatgtgcagctccatcagcaaaaggggatgataagtttatcaccaccgactatttgcaacagtgccgttgatcgtgctatgatcgactgttatctctggcggtgttgacactggagcacctcaaaaacaccatcatacactaaatcagtaagttggcagcatcacccgacgcactttgcgccgaataaatacctgtgacggaagatcacttcgcagaataaataaatcctggtgtccctgttgataccgggaagccctgggccaacttttggcgaaaatgagacgttgatcggcacgtaagaggttccaactttcaccataatgaaataagatcactaccgggcgtattttttgagttatcgagattttcaggagctaaggaagctaaaatggagaaaaaaatcactggatataccaccgttgatatatcccaatggcatcgtaaagaacattttgaggcatttcagtcagttgctcaatgtacctataaccagaccgttcagctggatattacggcctttttaaagaccgtaaagaaaaataagcacaagttttatccggcctttattcacattcttgcccgcctgatgaatgctcatccggaattccgtatggcaatgaaagacggtgagctggtgatatgggatagtgttcacccttgttacaccgttttccatgagcaaactgaaacgttttcatcgctctggagtgaataccacgacgatttccggcagtttctacacatatattcgcaagatgtggcgtgttacggtgaaaacctggcctatttccctaaagggtttattgagaatatgtttttcgtctcagccaatccctgggtgagtttcaccagttttgatttaaacgtggccaatatggacaacttcttcgcccccgttttcaccatgggcaaatattatacgcaaggcgacaaggtgctgatgccgctggcgattcaggttcatcatgccgtctgtgatggcttccatgtcggcagaatgcttaatgaattacaacagtactgcgatgagtggcagggcggggcgtaaggagatattccggacaacctttctcctttctatgaagcattgcaggaacagaagcccgatctttctgcggtccgctttggcgcaatcggtattggtagtcgtgaatatgacaccttttgtggggctatcgataaactcgaggcagaactcaaagattccggtgcaaaacagacaggcgaaacactgaagatcaacattcttgatcacgacattccggaagatccggcagaagaatggctgggatcgtggattaatttactcaaataagtatacagatcgtgcgatctactgtggataactctgtcaggaagcttggatcaaccggtagttatccaaggaacaacggttgttcagtttttgagttgtgtataacccctcattctgatcccagcttatacggtccaggatcactagtccatcgaaccgaagtatggccaaagcagaaatgccagctaattacctgattgtcggcaggcgattcgttgagtacagattcgatgttacgacaaatgatttcccccatttcatgaaatggcgggctgctgcacatggcggcgagaatattgccttcaagctggcgaatctgccgttcttcggtgatatccgagaaagtcattaccaggttctgcagatgcgcgagcacgtcataaaccgggctgatagaggctttaatccagattttttcaccggtgcgcgtcaacagcagaaattcgtcctgatcgcgggcggttttccatagcaactgttgtaaacgaatgcggttatcggcagggaattcaggaatgttcaggagtgtatcgggctgcataccgctggcttcgctaatgcagtaaccaaacatttcggtaaatgcgcgattgcactgcacaatatggcgttccggatcgaggacaatcaccggtcggtcgagatggtcaacggcaataatcaattgtcgggtctgttctttttgcgccatttctacgctggcatcccgtaccagcgccaggtaataaactttcccctcggcgctcactttcgatagcgca

**Editing cassette 3**

aatcgcccctggtaaaagaaacactgatgcgaggcctgtgtttcaatctttaaatcagtaaacttcatacgcttgacggaaaaaccaggacgaaacctaaatatttgttgttaagctgcaatggaaacggtaaaagcggctagtatttaaagggatggatgacatctcagcgttgtcggaggagatatttcatgatgatacgtgagcggatagaagaaaaattaagggcggcgttccaacccgtattcctcgaagtagtggatgaaagctatcgtcacaatgtcccagccggctctgaaagccattttaaagttgtgctggtcagcgatcgttttacgggtgaacgttttctgaatcgtcatcgaatgatttacagtactttagcggaggaactctctactaccgttcatgcgctggctctgcatacttacactattaaggagtgggaagggttgcaggacaccgtctttgcctctcctccctgtcgtggagcaggaagcatcgcgtaaaaacgcatttgcaactgtcggcgcttttccagtatgttgctaaagattttatgaaaaacggcctgcgggccgttttgttttgtctggatttaccgacgtcaggtggcacttttcggggaaatgtgcgcggaacccccatttgtttatttttctaaatacattcaaatatgtatccgctcatgagacaataaccctgataaatgcttcaataatattgaaaaaggaagagtatgagtattcaacatttccgtgtcgcccttattcccttttttgcggcattttgccttcctgtttttgctcacccagaaacgctggtgaaagtaaaagatgctgaagatcagttgggtgcacgagtgggttacatcgaactggatctcaacagcggtaagatccttgagagttttcgccccgaagaacgttttccaatgatgagcacttttaaagttctgctatgtggcgcggtattatcccgtattgacgccgggcaagagcaactcggtcgccgcatacactattctcagaatgacttggttgagtactcaccagtcacagaaaagcatcttacggatggcatgacagtaagagaattatgcagtgctgccataaccatgagtgataacactgcggccaacttacttctgacaacgatcggaggaccgaaggagctaaccgcttttttgcacaacatgggggatcatgcactagtccatcgaaccgaagtatggtatccaaggaacaacggttgttcagtttttgagttgtgtataacccctcattctgatcccagcttatacggtccaggatcaccgatcattcacagttaatgatcctttccaggttgttgatcttaaaagccggatccttgttatccacagggcagtgcgatcctaataagagatcacaatagaacagatctctaaataaatagatcttctttttaatacccaggatcccaggtctttctcaagccgacaaagttgagtagaatccacggcccgggcttcaatccattttcataccgcgttatgcgaggcaatcaccatgttttatccggatccttttgacgtcatcatcattggcgggggtcatgcaggcaccgaggccgcgatggccgcggcgcgtatgggtcaacagactcaagcacgttttgttttgtctggattttgcgctttttgcccagcattcagacgaaaattgcccgggaattgtgaaaaaatacgcgacagcgcgcaataaccgttctcgactcataaaagtgatgccgctataatgccgcgtcctatttgaatgctttcgggatgattctggtaacagggaatgtgattgattataagaacatcccggttccgcgaagccaacaacctgtgcttgcggggtaagagttgaccgagcactgtgattttttgaggtaacaagatgcaagtttcagttgaaaccactcaaggccttggccgccgtgtaacgattactatcgctgctgacagcatcgagaccgctgttaaaagcgagctggtcaacgttgcgaaaaaagtacgtattgacggcttccgcaaaggcaaagtgccaatgaatatcgttgctcagcgttatggcgcgtctgtacgccaggacgttctgggtgacctgatgagccgtaacttcattgacgccatcattaaagaaaaaatcaatccggctggcgcaccgacttatgttccgggcgaatacaagctgggtgaagacttcacttactctgtagagtttg

**Editing cassette 4**

atcaatacggtctacagccatccgcagccattccagcaatgcagcaaattccttaatcgttatccgcactggaagattgaatataccgaaagtacgtctgcggcaatggaaaaggttgcacaggcaaaatcaccgcatgttgctgcgttgggaagcgaagctggcggcactttgtacggtttgcaggtactggagcgtattgaagcaaatcagcgacaaaacttcacccgatttgtggtgttggcgcgtaaagccattaacgtgtctgatcaggttccggcgaaaaccacgttgttaatggcgaccgggcaacaagccggtgcgctggttgaagcgttgctggtactgcgcaaccacaatctgattatgacccgtctggaatcacgcccgattcacggtaatccatgggaagagatgttctatctggatattcaggccaatcttgaatcagcggaaatgcaaaaagcattgaaagagttaggggaaatcacccgttcaatgaaggtattgggctgttacccaagtgagaacgtagtgcctgttgatccaacctgatgaaaaggtgccggatgatgtgaatcatccggcactggattattaaccggttgacaattaatcatcggcatagtatatcggcatagtataatacgacaaggtgaggaactaaaccatggccaagttgaccagtgccgttccggtgctcaccgcgcgcgacgtcgccggagcggtcgagttctggaccgaccggctcgggttctcccgggacttcgtggaggacgacttcgccggtgtggtccgggacgacgtgaccctgttcatcagcgcggtccaggaccaggtggtgccggacaacaccctggcctgggtgtgggtgcgcggcctggacgagctgtacgccgagtggtcggaggtcgtgtccacgaacttccgggacgcctccgggccggccatgaccgagatcggcgagcagccgtgggggcgggagttcgccctgcgcgacccggccggcaactgcgtgcacttcgtggccgaggagcaggactgacacgtccgacggcggcccacgggtcccaggcctcggagatccgtcccccttttcctttgtcgatatcatgtaattagttatgtcacgcttacattcacgccctccccccacatccgctctaaccgaaaaggaaggagttagacaacctgaagtctaggtccctatttatttttttatagttatgttagtattaagaacgttatttatatttcaaatttttcttttttttctgtacagacgcgtgtacgcatgtaacattatactgaaaaccttgcttgagaaggttttgggacgctcgaaggctttaatttgcgcacagatattccggacaacctttctcctttctatgaagcattgcaggaacagaagcccgatctttctgcggtccgctttggcgcaatcggtattggtagtcgtgaatatgacaccttttgtggggctatcgataaactcgaggcagaactcaaagattccggtgcaaaacagacaggcgaaacactgaagatcaacattcttgatcacgacattccggaagatccggcagaagaatggctgggatcgtggattaatttactcaaataagtatacagatcgtgcgatctactgtggataactctgtcaggaagcttggatcaaccggtagttatccaaggaacaacggttgttcagtttttgagttgtgtataacccctcattctgatcccagcttatacggtccaggatcactagtccatcgaaccgaagtatggaccggaaggagctaaccgcttttttgcacaacatgggggatcatgtaactcgccttgatcgttgggaaccggagctgaatgaagccataccaaacgacgagcgtgacaccacgatgcctgtagcaatggcaacaacgttgcgcaaactattaactggcgaactacttactctagcttcccggcaacaattaatagactggatggaggcggataaagttgcaggaccacttctgcgctcggcccttccggctggctggtttattgctgataaatctggagccggtgagcgtgggtctcgcggtatcattgcagcactggggccagatggtaagccctcccgtatcgtagttatctacacgacggggagtcaggcaactatggatgaacgaaatagacagatcgctgagataggtgcctcactgattaagcattggtaactgtcagaccaagtttactcatatatactttagattgatttaaaacttcatttttaatttaaaaggatctaggtgaagatcctttttgataatctcatgaccaaaatcccttaacgtgagttttcgttccactgagcgtcagaccccgtagaaaagatcaaaggatcttcgcaccatccggcactggattattactggcgattgtcattcgcctgacgcaataacacgcggctttcactctgaaaacgctgtgcgtaatcgccgaaccagtgctccaccttgcggaaactgtcaataaacgcctgcttatcgccctgctccagcaactcaatcgcctcgccgaaacgcttatagtaacgtttgattaacgccagattacgctctgacgacataatgatgtcggcataaagctgcggatcctgagcaaacagtcgcccgaccatcgccagctcaaggcggtaaatcggcgaagagagcgccagaagttgctcaagctgaacattttcttctgccaggtgcagcccgtaagcaaaagtagcaaagtggcgcagtgcctgaataaacgccatattctgatcgtgctcgacggcgctaatacgatgcagccgagcgccccagacctgaatttgctccagaaaccattggtatgcttccggtttacgtccatcacaccagaccacaacttgctttgccaggctaccgctgtccggaccgaacatcgggtgtagccccagcaccggaccatcatgcgccaccagcatggcctgtaatggc

**Table S4. The sequences of clone PCR products used to identity the *E. coli* variants.**

**f_1_/r_1_ (*E. coli*^WT^): 931 bp**

ttggcggcttgaatattgaacaggaggcgttatgccaatttatcagatagacggtctgactccggttgtgccagaagagagttttgtccatccgacagcggtattgatcggcgatgttattctcggcaagggcgtttacgttgggccaaatgccagcctgcgtggcgattttggtcgtatcgtggtgaaagatggcgcgaacattcaggataattgcgttatgcacggttttcccgagcaggatactgttgtaggagaagatggacatattggtcatagcgctatccttcacggctgcattatccgccgcaatgcattagtgggaatgaacgcggtagtgatggacggtgcggtgattggcgagaacagcattgttggtgcatccgcatttgtgaaagccaaagcagaaatgccagctaattacctgattgtcggcagcccggcgaaagcgattcgtgaactcagtgagcaggagttggcatggaaaaagcagggtacgcatgagtaccaggtgctggtgacacgctgtaagcagacgttacatcaagtcgagccattgcgggaaattgaacctggcaggaaacgcctggtatttgatgagaatctgcgaccgaaacagtaacagatgtaaaattattttgtccctttaattataaagcagagttatgtttaagctctgctttatttatttgagtattaattcataccgttttttcatcatataattatttataatgagaatgtggttttaatttgtaatttatattatatacacaatttatatatttcatggtctttttttattcacctgaattataattgtgaattataggaaagtatgtttgattagataataatctactggcaatattggatgtcttctatgttttaaataactaattggtcgggttagtgcatccggctttcttta

**f_1_/r_1_ (*E. coli*^C1/C2^): 1320 bp**

ttggcggcttgaatattgaacaggaggcgttatgccaatttatcagatagacggtctgactccggttgtgccagaagagagttttgtccatccgacagcggtattgatcggcgatgttattctcggcaagggcgtttacgttgggccaaatgccagcctgcgtggcgattttggtcgtatcgtggtgaaagatggcgcgaacattcaggataattgcgttatgcacggttttcccgagcaggatactgttgtaggagaagatggacatattggtcatagcgctatccttcacggctgcattatccgccgcaatgcattagtgggaatgaacgcggtagtgatggacggtgcggtgattggcgagaacagcattgttggtgcatccgcatttgtgaaagccaaagcagaaatgccagctaattacctgattgtcggcagtagtccatcgaaccgaagtatggtatccaaggaacaacggttgttcagtttttgagttgtgtataacccctcattctgatcccagcttatacggtccaggatcaccgatcattcacagttaatgatcctttccaggttgttgatcttaaaagccggatccttgttatccacagggcagtgcgatcctaataagagatcacaatagaacagatctctaaataaatagatcttctttttaatacccaggatcccaggtctttctcaagccgacaaagttgagtagaatccacggcccgggcttcaatccattttcataccgcgttatgcgaggcaatcaccatgttttatccggatccttttgacgtcatcatcattggcgggggtcatgcaggcaccgaggccgcgatggccgcggcgcgtatgggtcaacagactcaaaagcagggtacgcatgagtaccaggtgctggtgacacgctgtaagcagacgttacatcaagtcgagccattgcgggaaattgaacctggcaggaaacgcctggtatttgatgagaatctgcgaccgaaacagtaacagatgtaaaattattttgtccctttaattataaagcagagttatgtttaagctctgctttatttatttgagtattaattcataccgttttttcatcatataattatttataatgagaatgtggttttaatttgtaatttatattatatacacaatttatatatttcatggtctttttttattcacctgaattataattgtgaattataggaaagtatgtttgattagataataatctactggcaatattggatgtcttctatgttttaaataactaattggtcgggttagtgcatccggctttcttta

**f_2_/r_2_ (*E. coli*^WT^): 2905 bp**

ccaccagtcattatcgcattcagcaacagccgattgcgctgagcggcgggcgggacggaagggtgataggcatgggagcgctcgtgcatttcatcgaaccctgcgcccatatcgaggatgttcccgtgctcatcacgaagcgtcaggtcgatcgccgtgccacggctgtgattagaaccgaccgtcacatcaacaacatattgcgggtctgggcaggcttgccacaacatcgcctgtgcttgttgtgggcgatacgcatcgtaaatcaccagttgtaaccctgacagctgggcgatgctgatacttttcgccagcgcggtaatcgcatccttgtgtaacagacaacgcgcttgctgataaatagctttacctgtgatgttatcagcgcaggcgtatttcaattcgatctccagatcagggaagattacggctaaatcaaccagttcggtggtatccgacataaacggttcctgtataagacaaaaattgctgcgctttccgcttatgcagatctcatgccatgccgggataagcgccagaatgctggcttaaagttatattttttaacttttgatcaacatttgtgcagcgtagtgcagttttggtgcaagaggggaagttaaggaaggaatctcccggaatcgtagctgaaatcacagtatttaagtgacagtgtcacgttaaatgaaaacccgcgagtgcgggcgagaggaatttgtcagattttcagcggtaacacgctgctcatccagcctggaatttcttcggcgggtagggggcgggaaaagaaatatccctgaataacgcgacagtggatcttgcgtagcatctcaaattgctctttggtttcgacgccttccgccacgacggttaaattgaggctttgcccaatgctggtaatggcttcaagtaaggcaaggatgcgtttttcggtcagacaacgatcgacaaaacttttgtcaattttgatttccgttaccggaagactgactaagcgggataatccggaaaagcccgtaccaaaatcatctaccgataagcccacgcccatatcacgcaggatctgaatgcgcttaaagatttcggtatcgtgttccatcatcatgctttccgtgatttctaccgtcagctggtggccgtcaataccccaggcgtgcattgcatcagacacctgattaggcagttgattactgcgaaagtgcagcgccgacaagttcacggataacgccgggatatgaatattctggctacgccattctgctaactgacggcaagcttccgcgatgacccagcgcccaatattttcgatttcaccaatctcttctgcgagaggaataaaccgtgaagggggcacatgaccatgcaggggatcgtgccagcgagcaagggcttcgatgccgtacagttcacccgtttctgcgaagatttgcggctggtaaaccagtttcagttggttattgctaatcgcttctttcagcgctgcgcctaaaaccaaacgctcttttaccatttcgttcatcgccgggctgaagaactgccagccgttaccgccattcttgcgaatataatccattgcattgtgagcagtggagagcaagtaatcgcggtttttacccaggtcgtagctgatgccaatactcaaggtaagcgggaagggtttatcgtcaatcattatcggcttgctgaccacattccgtagctcatcggcgatttgggtaatgttactgacgtcgttttcgaggctcacgaggacaaactgcgtaccttcgatacgacagagatactgatccggtttgagtttttcacgaaagcgattgaccacttccagcaatgcctgatcggcccacgcatagccaaggctatcaatcacatcctgaatatggtcaacaccgatgagatacaccacgggagagacggctttgtcgaccaggtcatcgaggtaattgtgcaggttattgcgatttggcagaccggtcatcggatcaaattggatgagttgttcaatatgctgacggcttttttcctgttccagcgccagcgcggccatatgctggctgatatctgccacgcgttcgataaaggcgctggtttctgctcctgacgaggttttaatttgcaggatccccgcaggcgcgccatcacgctgacgaatggtcgctgaccagctttgcgcattttgaatttctgcaccgtgggaagatgacgcccagtgtatcggcatcccgttgcgcagtgcgaacagcgaaacatgcgattcgttgagtacagattcgatgttacgacaaatgatttcccccatttcatgaaatggcgggctgctgcacatggcggcgagaatattgccttcaagctggcgaatctgccgttcttcggtgatatccgagaaagtcattaccaggttctgcagatgcgcgagcacgtcataaaccgggctgatagaggctttaatccagattttttcaccggtgcgcgtcaacagcagaaattcgtcctgatcgcgggcggttttccatagcaactgttgtaaacgaatgcggttatcggcagggaattcaggaatgttcaggagtgtatcgggctgcataccgctggcttcgctaatgcagtaaccaaacatttcggtaaatgcgcgattgcactgcacaatatggcgttccggatcgaggacaatcaccggtcggtcgagatggtcaacggcaataatcaattgtcgggtctgttctttttgcgccatttctacgctggcatcccgtaccagcgccaggtaataaactttcccctcggcgctcactttcgatagcgcaaaacgggtccagattttactgccgtcttttttctccagctgcagctcccgactcatcccctcaacacgcgctttaccgccttcacggttgtgacgaatgta

**f_2_/r_2_ (*E. coli*^C1/C2^): 2956 bp**

ccaccagtcattatcgcattcagcaacagccgattgcgctgagcggcgggcgggacggaagggtgataggcatgggagcgctcgtgcatttcatcgaaccctgcgcccatatcgaggatgttcccgtgctcatcacgaagcgtcaggtcgatcgccgtgccacggctgtgattagaaccgaccgtcacatcaacaacatattgcgggtctgggcaggcttgccacaacatcgcctgtgcttgttgtgggcgatacgcatcgtaaatcaccagttgtaaccctgacagctgggcgatgctgatacttttcgccagcgcggtaatcgcatccttgtgtaacagacaacgcgcttgctgataaatagctttacctgtgatgttatcagcgcaggcgtatttcaattcgatctccagatcagggaagattacggctaaatcaaccagttcggtggtatccgacataaacggttcctgtataagacaaaaattgctgcgctttccgcttatgcagatctcatgccatgccgggataagcgccagaatgctggcttaaagttatattttttaacttttgatcaacatttgtgcagcgtagtgcagttttggtgcaagaggggaagttaaggaaggaatctcccggaatcgtagctgaaatcacagtatttaagtgacagtgtcacgttaaatgaaaacccgcgagtgcgggcgagaggaatttgtcagattttcagcggtaacacgctgctcatccagcctggaatttcttcggcgggtaccagcatgtgcagctccatcagcaaaaggggatgataagtttatcaccaccgactatttgcaacagtgccgttgatcgtgctatgatcgactgttatctctggcggtgttgacactggagcacctcaaaaacaccatcatacactaaatcagtaagttggcagcatcacccgacgcactttgcgccgaataaatacctgtgacggaagatcacttcgcagaataaataaatcctggtgtccctgttgataccgggaagccctgggccaacttttggcgaaaatgagacgttgatcggcacgtaagaggttccaactttcaccataatgaaataagatcactaccgggcgtattttttgagttatcgagattttcaggagctaaggaagctaaaatggagaaaaaaatcactggatataccaccgttgatatatcccaatggcatcgtaaagaacattttgaggcatttcagtcagttgctcaatgtacctataaccagaccgttcagctggatattacggcctttttaaagaccgtaaagaaaaataagcacaagttttatccggcctttattcacattcttgcccgcctgatgaatgctcatccggaattccgtatggcaatgaaagacggtgagctggtgatatgggatagtgttcacccttgttacaccgttttccatgagcaaactgaaacgttttcatcgctctggagtgaataccacgacgatttccggcagtttctacacatatattcgcaagatgtggcgtgttacggtgaaaacctggcctatttccctaaagggtttattgagaatatgtttttcgtctcagccaatccctgggtgagtttcaccagttttgatttaaacgtggccaatatggacaacttcttcgcccccgttttcaccatgggcaaatattatacgcaaggcgacaaggtgctgatgccgctggcgattcaggttcatcatgccgtctgtgatggcttccatgtcggcagaatgcttaatgaattacaacagtactgcgatgagtggcagggcggggcgtaaggagatattccggacaacctttctcctttctatgaagcattgcaggaacagaagcccgatctttctgcggtccgctttggcgcaatcggtattggtagtcgtgaatatgacaccttttgtggggctatcgataaactcgaggcagaactcaaagattccggtgcaaaacagacaggcgaaacactgaagatcaacattcttgatcacgacattccggaagatccggcagaagaatggctgggatcgtggattaatttactcaaataagtatacagatcgtgcgatctactgtggataactctgtcaggaagcttggatcaaccggtagttatccaaggaacaacggttgttcagtttttgagttgtgtataacccctcattctgatcccagcttatacggtccaggatcactagtccatcgaaccgaagtatggccaaagcagaaatgccagctaattacctgattgtcggcaggcgattcgttgagtacagattcgatgttacgacaaatgatttcccccatttcatgaaatggcgggctgctgcacatggcggcgagaatattgccttcaagctggcgaatctgccgttcttcggtgatatccgagaaagtcattaccaggttctgcagatgcgcgagcacgtcataaaccgggctgatagaggctttaatccagattttttcaccggtgcgcgtcaacagcagaaattcgtcctgatcgcgggcggttttccatagcaactgttgtaaacgaatgcggttatcggcagggaattcaggaatgttcaggagtgtatcgggctgcataccgctggcttcgctaatgcagtaaccaaacatttcggtaaatgcgcgattgcactgcacaatatggcgttccggatcgaggacaatcaccggtcggtcgagatggtcaacggcaataatcaattgtcgggtctgttctttttgcgccatttctacgctggcatcccgtaccagcgccaggtaataaactttcccctcggcgctcactttcgatagcgcaaaacgggtccagattttactgccgtcttttttctccagctgcagctcccgactcatcccctcaacacgcgctttaccgccttcacggttgtgacgaatgta

**f_1_/r_2_ (*E. coli*^0.10/4.54^): 1093 bp** ttggcggcttgaatattgaacaggaggcgttatgccaatttatcagatagacggtctgactccggttgtgccagaagagagttttgtccatccgacagcggtattgatcggcgatgttattctcggcaagggcgtttacgttgggccaaatgccagcctgcgtggcgattttggtcgtatcgtggtgaaagatggcgcgaacattcaggataattgcgttatgcacggttttcccgagcaggatactgttgtaggagaagatggacatattggtcatagcgctatccttcacggctgcattatccgccgcaatgcattagtgggaatgaacgcggtagtgatggacggtgcggtgattggcgagaacagcattgttggtgcatccgcatttgtgaaagccaaagcagaaatgccagctaattacctgattgtcggcaggcgattcgttgagtacagattcgatgttacgacaaatgatttcccccatttcatgaaatggcgggctgctgcacatggcggcgagaatattgccttcaagctggcgaatctgccgttcttcggtgatatccgagaaagtcattaccaggttctgcagatgcgcgagcacgtcataaaccgggctgatagaggctttaatccagattttttcaccggtgcgcgtcaacagcagaaattcgtcctgatcgcgggcggttttccatagcaactgttgtaaacgaatgcggttatcggcagggaattcaggaatgttcaggagtgtatcgggctgcataccgctggcttcgctaatgcagtaaccaaacatttcggtaaatgcgcgattgcactgcacaatatggcgttccggatcgaggacaatcaccggtcggtcgagatggtcaacggcaataatcaattgtcgggtctgttctttttgcgccatttctacgctggcatcccgtaccagcgccaggtaataaactttcccctcggcgctcactttcgatagcgcaaaacgggtccagattttactgccgtcttttttctccagctgcagctcccgactcatcccctcaacacgcgctttaccgccttcacggttgtgacgaatgta

**f_2_/r_1_ (*E. coli*^0.10/4.54^): 3015 bp** ccaccagtcattatcgcattcagcaacagccgattgcgctgagcggcgggcgggacggaagggtgataggcatgggagcgctcgtgcatttcatcgaaccctgcgcccatatcgaggatgttcccgtgctcatcacgaagcgtcaggtcgatcgccgtgccacggctgtgattagaaccgaccgtcacatcaacaacatattgcgggtctgggcaggcttgccacaacatcgcctgtgcttgttgtgggcgatacgcatcgtaaatcaccagttgtaaccctgacagctgggcgatgctgatacttttcgccagcgcggtaatcgcatccttgtgtaacagacaacgcgcttgctgataaatagctttacctgtgatgttatcagcgcaggcgtatttcaattcgatctccagatcagggaagattacggctaaatcaaccagttcggtggtatccgacataaacggttcctgtataagacaaaaattgctgcgctttccgcttatgcagatctcatgccatgccgggataagcgccagaatgctggcttaaagttatattttttaacttttgatcaacatttgtgcagcgtagtgcagttttggtgcaagaggggaagttaaggaaggaatctcccggaatcgtagctgaaatcacagtatttaagtgacagtgtcacgttaaatgaaaacccgcgagtgcgggcgagaggaatttgtcagattttcagcggtaacacgctgctcatccagcctggaatttcttcggcgggtaccagcatgtgcagctccatcagcaaaaggggatgataagtttatcaccaccgactatttgcaacagtgccgttgatcgtgctatgatcgactgttatctctggcggtgttgacactggagcacctcaaaaacaccatcatacactaaatcagtaagttggcagcatcacccgacgcactttgcgccgaataaatacctgtgacggaagatcacttcgcagaataaataaatcctggtgtccctgttgataccgggaagccctgggccaacttttggcgaaaatgagacgttgatcggcacgtaagaggttccaactttcaccataatgaaataagatcactaccgggcgtattttttgagttatcgagattttcaggagctaaggaagctaaaatggagaaaaaaatcactggatataccaccgttgatatatcccaatggcatcgtaaagaacattttgaggcatttcagtcagttgctcaatgtacctataaccagaccgttcagctggatattacggcctttttaaagaccgtaaagaaaaataagcacaagttttatccggcctttattcacattcttgcccgcctgatgaatgctcatccggaattccgtatggcaatgaaagacggtgagctggtgatatgggatagtgttcacccttgttacaccgttttccatgagcaaactgaaacgttttcatcgctctggagtgaataccacgacgatttccggcagtttctacacatatattcgcaagatgtggcgtgttacggtgaaaacctggcctatttccctaaagggtttattgagaatatgtttttcgtctcagccaatccctgggtgagtttcaccagttttgatttaaacgtggccaatatggacaacttcttcgcccccgttttcaccatgggcaaatattatacgcaaggcgacaaggtgctgatgccgctggcgattcaggttcatcatgccgtctgtgatggcttccatgtcggcagaatgcttaatgaattacaacagtactgcgatgagtggcagggcggggcgtaaggagatattccggacaacctttctcctttctatgaagcattgcaggaacagaagcccgatctttctgcggtccgctttggcgcaatcggtattggtagtcgtgaatatgacaccttttgtggggctatcgataaactcgaggcagaactcaaagattccggtgcaaaacagacaggcgaaacactgaagatcaacattcttgatcacgacattccggaagatccggcagaagaatggctgggatcgtggattaatttactcaaataagtatacagatcgtgcgatctactgtggataactctgtcaggaagcttggatcaaccggtagttatccaaggaacaacggttgttcagtttttgagttgtgtataacccctcattctgatcccagcttatacggtccaggatcaccgatcattcacagttaatgatcctttccaggttgttgatcttaaaagccggatccttgttatccacagggcagtgcgatcctaataagagatcacaatagaacagatctctaaataaatagatcttctttttaatacccaggatcccaggtctttctcaagccgacaaagttgagtagaatccacggcccgggcttcaatccattttcataccgcgttatgcgaggcaatcaccatgttttatccggatccttttgacgtcatcatcattggcgggggtcatgcaggcaccgaggccgcgatggccgcggcgcgtatgggtcaacagactcaaaagcagggtacgcatgagtaccaggtgctggtgacacgctgtaagcagacgttacatcaagtcgagccattgcgggaaattgaacctggcaggaaacgcctggtatttgatgagaatctgcgaccgaaacagtaacagatgtaaaattattttgtccctttaattataaagcagagttatgtttaagctctgctttatttatttgagtattaattcataccgttttttcatcatataattatttataatgagaatgtggttttaatttgtaatttatattatatacacaatttatatatttcatggtctttttttattcacctgaattataattgtgaattataggaaagtatgtttgattagataataatctactggcaatattggatgtcttctatgttttaaataactaattggtcgggttagtgcatccggctttcttta

**f_3_/r_3_ (*E. coli*^WT^): 1917 bp**

ttgcagattaaccgggccattcggaccaaccatgtaaccacgcgcggtcatctgtttttccagcacttcttgcagcaggaaacgcagatcgcgggaggcggtcagggtaacgatttgattatcgcgggtgacttttgccagcgcctgatcggtacgctgatcggcaccattaatgcttacggtgacgcccatcaggcttggatcctgctgtggcagtgtaatcgtcggggaaacttcaatagttgttggcggttttgcgcatcctgcaagcataaacagagcaactaacgggaagaggatttttttgaacatgttcgggctctcagagactcttaagcgtgtttggtaaaaattcccgccatcataacattgccaacggcgaggggaagtgggtaaggcatgtaaattcatcatgttgacgaaataatcgcccctggtaaaagaaacactgatgcgaggcctgtgtttcaatctttaaatcagtaaacttcatacgcttgacggaaaaaccaggacgaaacctaaatatttgttgttaagctgcaatggaaacggtaaaagcggctagtatttaaagggatggatgacatctcagcgttgtcggaggagatatttcatgatgatacgtgagcggatagaagaaaaattaagggcggcgttccaacccgtattcctcgaagtagtggatgaaagctatcgtcacaatgtcccagccggctctgaaagccattttaaagttgtgctggtcagcgatcgttttacgggtgaacgttttctgaatcgtcatcgaatgatttacagtactttagcggaggaactctctactaccgttcatgcgctggctctgcatacttacactattaaggagtgggaagggttgcaggacaccgtctttgcctctcctccctgtcgtggagcaggaagcatcgcgtaaaaacgcatttgcaactgtcggcgcttttccagtatgttgctaaagattttatgaaaaacggcctgcgggccgttttgttttgtctggattttgcgctttttgcccagcattcagacgaaaattgcccgggaattgtgaaaaaatacgcgacagcgcgcaataaccgttctcgactcataaaagtgatgccgctataatgccgcgtcctatttgaatgctttcgggatgattctggtaacagggaatgtgattgattataagaacatcccggttccgcgaagccaacaacctgtgcttgcggggtaagagttgaccgagcactgtgattttttgaggtaacaagatgcaagtttcagttgaaaccactcaaggccttggccgccgtgtaacgattactatcgctgctgacagcatcgagaccgctgttaaaagcgagctggtcaacgttgcgaaaaaagtacgtattgacggcttccgcaaaggcaaagtgccaatgaatatcgttgctcagcgttatggcgcgtctgtacgccaggacgttctgggtgacctgatgagccgtaacttcattgacgccatcattaaagaaaaaatcaatccggctggcgcaccgacttatgttccgggcgaatacaagctgggtgaagacttcacttactctgtagagtttgaagtttatccggaagttgaactgcagggtctggaagcgatcgaagttgaaaaaccgatcgttgaagtgaccgacgctgacgttgacggcatgctggatactctgcgtaaacagcaggcgacctggaaagaaaaagacggcgctgttgaagcagaagaccgcgtaaccatcgacttcaccggttctgtagacggcgaagagttcgaaggcggtaaagcgtctgatttcgtactggcgatgggccagggtcgtatgatcccgggctttgaagacggtatcaaaggccacaaagctggcgaagagttcaccatcg

**f_3_/r_3_ (*E. coli*^C3/C4^): 2990 bp**

ttgcagattaaccgggccattcggaccaaccatgtaaccacgcgcggtcatctgtttttccagcacttcttgcagcaggaaacgcagatcgcgggaggcggtcagggtaacgatttgattatcgcgggtgacttttgccagcgcctgatcggtacgctgatcggcaccattaatgcttacggtgacgcccatcaggcttggatcctgctgtggcagtgtaatcgtcggggaaacttcaatagttgttggcggttttgcgcatcctgcaagcataaacagagcaactaacgggaagaggatttttttgaacatgttcgggctctcagagactcttaagcgtgtttggtaaaaattcccgccatcataacattgccaacggcgaggggaagtgggtaaggcatgtaaattcatcatgttgacgaaataatcgcccctggtaaaagaaacactgatgcgaggcctgtgtttcaatctttaaatcagtaaacttcatacgcttgacggaaaaaccaggacgaaacctaaatatttgttgttaagctgcaatggaaacggtaaaagcggctagtatttaaagggatggatgacatctcagcgttgtcggaggagatatttcatgatgatacgtgagcggatagaagaaaaattaagggcggcgttccaacccgtattcctcgaagtagtggatgaaagctatcgtcacaatgtcccagccggctctgaaagccattttaaagttgtgctggtcagcgatcgttttacgggtgaacgttttctgaatcgtcatcgaatgatttacagtactttagcggaggaactctctactaccgttcatgcgctggctctgcatacttacactattaaggagtgggaagggttgcaggacaccgtctttgcctctcctccctgtcgtggagcaggaagcatcgcgtaaaaacgcatttgcaactgtcggcgcttttccagtatgttgctaaagattttatgaaaaacggcctgcgggccgttttgttttgtctggatttaccgacgtcaggtggcacttttcggggaaatgtgcgcggaacccccatttgtttatttttctaaatacattcaaatatgtatccgctcatgagacaataaccctgataaatgcttcaataatattgaaaaaggaagagtatgagtattcaacatttccgtgtcgcccttattcccttttttgcggcattttgccttcctgtttttgctcacccagaaacgctggtgaaagtaaaagatgctgaagatcagttgggtgcacgagtgggttacatcgaactggatctcaacagcggtaagatccttgagagttttcgccccgaagaacgttttccaatgatgagcacttttaaagttctgctatgtggcgcggtattatcccgtattgacgccgggcaagagcaactcggtcgccgcatacactattctcagaatgacttggttgagtactcaccagtcacagaaaagcatcttacggatggcatgacagtaagagaattatgcagtgctgccataaccatgagtgataacactgcggccaacttacttctgacaacgatcggaggaccgaaggagctaaccgcttttttgcacaacatgggggatcatgcactagtccatcgaaccgaagtatggtatccaaggaacaacggttgttcagtttttgagttgtgtataacccctcattctgatcccagcttatacggtccaggatcaccgatcattcacagttaatgatcctttccaggttgttgatcttaaaagccggatccttgttatccacagggcagtgcgatcctaataagagatcacaatagaacagatctctaaataaatagatcttctttttaatacccaggatcccaggtctttctcaagccgacaaagttgagtagaatccacggcccgggcttcaatccattttcataccgcgttatgcgaggcaatcaccatgttttatccggatccttttgacgtcatcatcattggcgggggtcatgcaggcaccgaggccgcgatggccgcggcgcgtatgggtcaacagactcaagcacgttttgttttgtctggattttgcgctttttgcccagcattcagacgaaaattgcccgggaattgtgaaaaaatacgcgacagcgcgcaataaccgttctcgactcataaaagtgatgccgctataatgccgcgtcctatttgaatgctttcgggatgattctggtaacagggaatgtgattgattataagaacatcccggttccgcgaagccaacaacctgtgcttgcggggtaagagttgaccgagcactgtgattttttgaggtaacaagatgcaagtttcagttgaaaccactcaaggccttggccgccgtgtaacgattactatcgctgctgacagcatcgagaccgctgttaaaagcgagctggtcaacgttgcgaaaaaagtacgtattgacggcttccgcaaaggcaaagtgccaatgaatatcgttgctcagcgttatggcgcgtctgtacgccaggacgttctgggtgacctgatgagccgtaacttcattgacgccatcattaaagaaaaaatcaatccggctggcgcaccgacttatgttccgggcgaatacaagctgggtgaagacttcacttactctgtagagtttgaagtttatccggaagttgaactgcagggtctggaagcgatcgaagttgaaaaaccgatcgttgaagtgaccgacgctgacgttgacggcatgctggatactctgcgtaaacagcaggcgacctggaaagaaaaagacggcgctgttgaagcagaagaccgcgtaaccatcgacttcaccggttctgtagacggcgaagagttcgaaggcggtaaagcgtctgatttcgtactggcgatgggccagggtcgtatgatcccgggctttgaagacggtatcaaaggccacaaagctggcgaagagttcaccatcg

**f_4_/r_4_ (*E. coli*^WT^):** **1756 bp**

gatcgtgaacgcgatttgctggaaagattaattacgctcggtaaagcgcaccatctggacgcccattacattactcgcctgttccagctcatcattgaagattccgtattaactcagcaggctttgctccaacaacatctcaataaaattaatccgcactcagcacgcatcgcttttctcggccccaaaggttcttattcccatcttgcggcgcgccagtatgctgcccgtcactttgagcaattcattgaaagtggctgcgccaaatttgccgatatttttaatcaggtggaaaccggccaggccgactatgccgtcgtaccgattgaaaataccagctccggtgccataaacgacgtttacgatctgctgcaacataccagcttgtcgattgttggcgagatgacgttaactatcgaccattgtttgttggtctccggcactactgatttatccaccatcaatacggtctacagccatccgcagccattccagcaatgcagcaaattccttaatcgttatccgcactggaagattgaatataccgaaagtacgtctgcggcaatggaaaaggttgcacaggcaaaatcaccgcatgttgctgcgttgggaagcgaagctggcggcactttgtacggtttgcaggtactggagcgtattgaagcaaatcagcgacaaaacttcacccgatttgtggtgttggcgcgtaaagccattaacgtgtctgatcaggttccggcgaaaaccacgttgttaatggcgaccgggcaacaagccggtgcgctggttgaagcgttgctggtactgcgcaaccacaatctgattatgacccgtctggaatcacgcccgattcacggtaatccatgggaagagatgttctatctggatattcaggccaatcttgaatcagcggaaatgcaaaaagcattgaaagagttaggggaaatcacccgttcaatgaaggtattgggctgttacccaagtgagaacgtagtgcctgttgatccaacctgatgaaaaggtgccggatgatgtgaatcatccggcactggattattactggcgattgtcattcgcctgacgcaataacacgcggctttcactctgaaaacgctgtgcgtaatcgccgaaccagtgctccaccttgcggaaactgtcaataaacgcctgcttatcgccctgctccagcaactcaatcgcctcgccgaaacgcttatagtaacgtttgattaacgccagattacgctctgacgacataatgatgtcggcataaagctgcggatcctgagcaaacagtcgcccgaccatcgccagctcaaggcggtaaatcggcgaagagagcgccagaagttgctcaagctgaacattttcttctgccaggtgcagcccgtaagcaaaagtagcaaagtggcgcagtgcctgaataaacgccatattctgatcgtgctcgacggcgctaatacgatgcagccgagcgccccagacctgaatttgctccagaaaccattggtatgcttccggtttacgtccatcacaccagaccacaacttgctttgccaggctaccgctgtccggaccgaacatcgggtgtagccccagcaccggaccatcatgcgccaccagcatggcctgtaatggcccatttttcactgatgccagatcgaccagaatacaatctttcggtaaaggcggtaatttgccaataacttgctcagtaacgtggattggcacactaacaatcaccattccggcatcg

**f_4_/r_4_ (*E. coli*^C3/C4^): 3581 bp**

gatcgtgaacgcgatttgctggaaagattaattacgctcggtaaagcgcaccatctggacgcccattacattactcgcctgttccagctcatcattgaagattccgtattaactcagcaggctttgctccaacaacatctcaataaaattaatccgcactcagcacgcatcgcttttctcggccccaaaggttcttattcccatcttgcggcgcgccagtatgctgcccgtcactttgagcaattcattgaaagtggctgcgccaaatttgccgatatttttaatcaggtggaaaccggccaggccgactatgccgtcgtaccgattgaaaataccagctccggtgccataaacgacgtttacgatctgctgcaacataccagcttgtcgattgttggcgagatgacgttaactatcgaccattgtttgttggtctccggcactactgatttatccaccatcaatacggtctacagccatccgcagccattccagcaatgcagcaaattccttaatcgttatccgcactggaagattgaatataccgaaagtacgtctgcggcaatggaaaaggttgcacaggcaaaatcaccgcatgttgctgcgttgggaagcgaagctggcggcactttgtacggtttgcaggtactggagcgtattgaagcaaatcagcgacaaaacttcacccgatttgtggtgttggcgcgtaaagccattaacgtgtctgatcaggttccggcgaaaaccacgttgttaatggcgaccgggcaacaagccggtgcgctggttgaagcgttgctggtactgcgcaaccacaatctgattatgacccgtctggaatcacgcccgattcacggtaatccatgggaagagatgttctatctggatattcaggccaatcttgaatcagcggaaatgcaaaaagcattgaaagagttaggggaaatcacccgttcaatgaaggtattgggctgttacccaagtgagaacgtagtgcctgttgatccaacctgatgaaaaggtgccggatgatgtgaatcatccggcactggattattaaccggttgacaattaatcatcggcatagtatatcggcatagtataatacgacaaggtgaggaactaaaccatggccaagttgaccagtgccgttccggtgctcaccgcgcgcgacgtcgccggagcggtcgagttctggaccgaccggctcgggttctcccgggacttcgtggaggacgacttcgccggtgtggtccgggacgacgtgaccctgttcatcagcgcggtccaggaccaggtggtgccggacaacaccctggcctgggtgtgggtgcgcggcctggacgagctgtacgccgagtggtcggaggtcgtgtccacgaacttccgggacgcctccgggccggccatgaccgagatcggcgagcagccgtgggggcgggagttcgccctgcgcgacccggccggcaactgcgtgcacttcgtggccgaggagcaggactgacacgtccgacggcggcccacgggtcccaggcctcggagatccgtcccccttttcctttgtcgatatcatgtaattagttatgtcacgcttacattcacgccctccccccacatccgctctaaccgaaaaggaaggagttagacaacctgaagtctaggtccctatttatttttttatagttatgttagtattaagaacgttatttatatttcaaatttttcttttttttctgtacagacgcgtgtacgcatgtaacattatactgaaaaccttgcttgagaaggttttgggacgctcgaaggctttaatttgcgcacagatattccggacaacctttctcctttctatgaagcattgcaggaacagaagcccgatctttctgcggtccgctttggcgcaatcggtattggtagtcgtgaatatgacaccttttgtggggctatcgataaactcgaggcagaactcaaagattccggtgcaaaacagacaggcgaaacactgaagatcaacattcttgatcacgacattccggaagatccggcagaagaatggctgggatcgtggattaatttactcaaataagtatacagatcgtgcgatctactgtggataactctgtcaggaagcttggatcaaccggtagttatccaaggaacaacggttgttcagtttttgagttgtgtataacccctcattctgatcccagcttatacggtccaggatcactagtccatcgaaccgaagtatggaccggaaggagctaaccgcttttttgcacaacatgggggatcatgtaactcgccttgatcgttgggaaccggagctgaatgaagccataccaaacgacgagcgtgacaccacgatgcctgtagcaatggcaacaacgttgcgcaaactattaactggcgaactacttactctagcttcccggcaacaattaatagactggatggaggcggataaagttgcaggaccacttctgcgctcggcccttccggctggctggtttattgctgataaatctggagccggtgagcgtgggtctcgcggtatcattgcagcactggggccagatggtaagccctcccgtatcgtagttatctacacgacggggagtcaggcaactatggatgaacgaaatagacagatcgctgagataggtgcctcactgattaagcattggtaactgtcagaccaagtttactcatatatactttagattgatttaaaacttcatttttaatttaaaaggatctaggtgaagatcctttttgataatctcatgaccaaaatcccttaacgtgagttttcgttccactgagcgtcagaccccgtagaaaagatcaaaggatcttcgcaccatccggcactggattattactggcgattgtcattcgcctgacgcaataacacgcggctttcactctgaaaacgctgtgcgtaatcgccgaaccagtgctccaccttgcggaaactgtcaataaacgcctgcttatcgccctgctccagcaactcaatcgcctcgccgaaacgcttatagtaacgtttgattaacgccagattacgctctgacgacataatgatgtcggcataaagctgcggatcctgagcaaacagtcgcccgaccatcgccagctcaaggcggtaaatcggcgaagagagcgccagaagttgctcaagctgaacattttcttctgccaggtgcagcccgtaagcaaaagtagcaaagtggcgcagtgcctgaataaacgccatattctgatcgtgctcgacggcgctaatacgatgcagccgagcgccccagacctgaatttgctccagaaaccattggtatgcttccggtttacgtccatcacaccagaccacaacttgctttgccaggctaccgctgtccggaccgaacatcgggtgtagccccagcaccggaccatcatgcgccaccagcatggcctgtaatggcccatttttcactgatgccagatcgaccagaatacaatctttcggtaaaggcggtaatttgccaataacttgctcagtaacgtggattggcacactaacaatcaccattccggcatcg

**f_3_/r_4_ (*E. coli*^2.28/2.36^)：2916 bp**

ttgcagattaaccgggccattcggaccaaccatgtaaccacgcgcggtcatctgtttttccagcacttcttgcagcaggaaacgcagatcgcgggaggcggtcagggtaacgatttgattatcgcgggtgacttttgccagcgcctgatcggtacgctgatcggcaccattaatgcttacggtgacgcccatcaggcttggatcctgctgtggcagtgtaatcgtcggggaaacttcaatagttgttggcggttttgcgcatcctgcaagcataaacagagcaactaacgggaagaggatttttttgaacatgttcgggctctcagagactcttaagcgtgtttggtaaaaattcccgccatcataacattgccaacggcgaggggaagtgggtaaggcatgtaaattcatcatgttgacgaaataatcgcccctggtaaaagaaacactgatgcgaggcctgtgtttcaatctttaaatcagtaaacttcatacgcttgacggaaaaaccaggacgaaacctaaatatttgttgttaagctgcaatggaaacggtaaaagcggctagtatttaaagggatggatgacatctcagcgttgtcggaggagatatttcatgatgatacgtgagcggatagaagaaaaattaagggcggcgttccaacccgtattcctcgaagtagtggatgaaagctatcgtcacaatgtcccagccggctctgaaagccattttaaagttgtgctggtcagcgatcgttttacgggtgaacgttttctgaatcgtcatcgaatgatttacagtactttagcggaggaactctctactaccgttcatgcgctggctctgcatacttacactattaaggagtgggaagggttgcaggacaccgtctttgcctctcctccctgtcgtggagcaggaagcatcgcgtaaaaacgcatttgcaactgtcggcgcttttccagtatgttgctaaagattttatgaaaaacggcctgcgggccgttttgttttgtctggatttaccgacgtcaggtggcacttttcggggaaatgtgcgcggaacccccatttgtttatttttctaaatacattcaaatatgtatccgctcatgagacaataaccctgataaatgcttcaataatattgaaaaaggaagagtatgagtattcaacatttccgtgtcgcccttattcccttttttgcggcattttgccttcctgtttttgctcacccagaaacgctggtgaaagtaaaagatgctgaagatcagttgggtgcacgagtgggttacatcgaactggatctcaacagcggtaagatccttgagagttttcgccccgaagaacgttttccaatgatgagcacttttaaagttctgctatgtggcgcggtattatcccgtattgacgccgggcaagagcaactcggtcgccgcatacactattctcagaatgacttggttgagtactcaccagtcacagaaaagcatcttacggatggcatgacagtaagagaattatgcagtgctgccataaccatgagtgataacactgcggccaacttacttctgacaacgatcggaggaccgaaggagctaaccgcttttttgcacaacatgggggatcatgtaactcgccttgatcgttgggaaccggagctgaatgaagccataccaaacgacgagcgtgacaccacgatgcctgtagcaatggcaacaacgttgcgcaaactattaactggcgaactacttactctagcttcccggcaacaattaatagactggatggaggcggataaagttgcaggaccacttctgcgctcggcccttccggctggctggtttattgctgataaatctggagccggtgagcgtgggtctcgcggtatcattgcagcactggggccagatggtaagccctcccgtatcgtagttatctacacgacggggagtcaggcaactatggatgaacgaaatagacagatcgctgagataggtgcctcactgattaagcattggtaactgtcagaccaagtttactcatatatactttagattgatttaaaacttcatttttaatttaaaaggatctaggtgaagatcctttttgataatctcatgaccaaaatcccttaacgtgagttttcgttccactgagcgtcagaccccgtagaaaagatcaaaggatcttcgcaccatccggcactggattattactggcgattgtcattcgcctgacgcaataacacgcggctttcactctgaaaacgctgtgcgtaatcgccgaaccagtgctccaccttgcggaaactgtcaataaacgcctgcttatcgccctgctccagcaactcaatcgcctcgccgaaacgcttatagtaacgtttgattaacgccagattacgctctgacgacataatgatgtcggcataaagctgcggatcctgagcaaacagtcgcccgaccatcgccagctcaaggcggtaaatcggcgaagagagcgccagaagttgctcaagctgaacattttcttctgccaggtgcagcccgtaagcaaaagtagcaaagtggcgcagtgcctgaataaacgccatattctgatcgtgctcgacggcgctaatacgatgcagccgagcgccccagacctgaatttgctccagaaaccattggtatgcttccggtttacgtccatcacaccagaccacaacttgctttgccaggctaccgctgtccggaccgaacatcgggtgtagccccagcaccggaccatcatgcgccaccagcatggcctgtaatggcccatttttcactgatgccagatcgaccagaatacaatctttcggtaaaggcggtaatttgccaataacttgctcagtaacgtggattggcacactaacaatcaccattccggcatcg

**f_4_/r_3_ (*E. coli*^2.28/2.36^)：3479 bp**

gatcgtgaacgcgatttgctggaaagattaattacgctcggtaaagcgcaccatctggacgcccattacattactcgcctgttccagctcatcattgaagattccgtattaactcagcaggctttgctccaacaacatctcaataaaattaatccgcactcagcacgcatcgcttttctcggccccaaaggttcttattcccatcttgcggcgcgccagtatgctgcccgtcactttgagcaattcattgaaagtggctgcgccaaatttgccgatatttttaatcaggtggaaaccggccaggccgactatgccgtcgtaccgattgaaaataccagctccggtgccataaacgacgtttacgatctgctgcaacataccagcttgtcgattgttggcgagatgacgttaactatcgaccattgtttgttggtctccggcactactgatttatccaccatcaatacggtctacagccatccgcagccattccagcaatgcagcaaattccttaatcgttatccgcactggaagattgaatataccgaaagtacgtctgcggcaatggaaaaggttgcacaggcaaaatcaccgcatgttgctgcgttgggaagcgaagctggcggcactttgtacggtttgcaggtactggagcgtattgaagcaaatcagcgacaaaacttcacccgatttgtggtgttggcgcgtaaagccattaacgtgtctgatcaggttccggcgaaaaccacgttgttaatggcgaccgggcaacaagccggtgcgctggttgaagcgttgctggtactgcgcaaccacaatctgattatgacccgtctggaatcacgcccgattcacggtaatccatgggaagagatgttctatctggatattcaggccaatcttgaatcagcggaaatgcaaaaagcattgaaagagttaggggaaatcacccgttcaatgaaggtattgggctgttacccaagtgagaacgtagtgcctgttgatccaacctgatgaaaaggtgccggatgatgtgaatcatccggcactggattattaaccggttgacaattaatcatcggcatagtatatcggcatagtataatacgacaaggtgaggaactaaaccatggccaagttgaccagtgccgttccggtgctcaccgcgcgcgacgtcgccggagcggtcgagttctggaccgaccggctcgggttctcccgggacttcgtggaggacgacttcgccggtgtggtccgggacgacgtgaccctgttcatcagcgcggtccaggaccaggtggtgccggacaacaccctggcctgggtgtgggtgcgcggcctggacgagctgtacgccgagtggtcggaggtcgtgtccacgaacttccgggacgcctccgggccggccatgaccgagatcggcgagcagccgtgggggcgggagttcgccctgcgcgacccggccggcaactgcgtgcacttcgtggccgaggagcaggactgacacgtccgacggcggcccacgggtcccaggcctcggagatccgtcccccttttcctttgtcgatatcatgtaattagttatgtcacgcttacattcacgccctccccccacatccgctctaaccgaaaaggaaggagttagacaacctgaagtctaggtccctatttatttttttatagttatgttagtattaagaacgttatttatatttcaaatttttcttttttttctgtacagacgcgtgtacgcatgtaacattatactgaaaaccttgcttgagaaggttttgggacgctcgaaggctttaatttgcgcacagatattccggacaacctttctcctttctatgaagcattgcaggaacagaagcccgatctttctgcggtccgctttggcgcaatcggtattggtagtcgtgaatatgacaccttttgtggggctatcgataaactcgaggcagaactcaaagattccggtgcaaaacagacaggcgaaacactgaagatcaacattcttgatcacgacattccggaagatccggcagaagaatggctgggatcgtggattaatttactcaaataagtatacagatcgtgcgatctactgtggataactctgtcaggaagcttggatcaaccggtagttatccaaggaacaacggttgttcagtttttgagttgtgtataacccctcattctgatcccagcttatacggtccaggatcaccgatcattcacagttaatgatcctttccaggttgttgatcttaaaagccggatccttgttatccacagggcagtgcgatcctaataagagatcacaatagaacagatctctaaataaatagatcttctttttaatacccaggatcccaggtctttctcaagccgacaaagttgagtagaatccacggcccgggcttcaatccattttcataccgcgttatgcgaggcaatcaccatgttttatccggatccttttgacgtcatcatcattggcgggggtcatgcaggcaccgaggccgcgatggccgcggcgcgtatgggtcaacagactcaagcacgttttgttttgtctggattttgcgctttttgcccagcattcagacgaaaattgcccgggaattgtgaaaaaatacgcgacagcgcgcaataaccgttctcgactcataaaagtgatgccgctataatgccgcgtcctatttgaatgctttcgggatgattctggtaacagggaatgtgattgattataagaacatcccggttccgcgaagccaacaacctgtgcttgcggggtaagagttgaccgagcactgtgattttttgaggtaacaagatgcaagtttcagttgaaaccactcaaggccttggccgccgtgtaacgattactatcgctgctgacagcatcgagaccgctgttaaaagcgagctggtcaacgttgcgaaaaaagtacgtattgacggcttccgcaaaggcaaagtgccaatgaatatcgttgctcagcgttatggcgcgtctgtacgccaggacgttctgggtgacctgatgagccgtaacttcattgacgccatcattaaagaaaaaatcaatccggctggcgcaccgacttatgttccgggcgaatacaagctgggtgaagacttcacttactctgtagagtttgaagtttatccggaagttgaactgcagggtctggaagcgatcgaagttgaaaaaccgatcgttgaagtgaccgacgctgacgttgacggcatgctggatactctgcgtaaacagcaggcgacctggaaagaaaaagacggcgctgttgaagcagaagaccgcgtaaccatcgacttcaccggttctgtagacggcgaagagttcgaaggcggtaaagcgtctgatttcgtactggcgatgggccagggtcgtatgatcccgggctttgaagacggtatcaaaggccacaaagctggcgaagagttcaccatcg

**f_5_/r_5_ (*E. coli*^2.28/2.36^)：3025 bp**

ctgcgccataaacctgtagcacaccattattgagcgtagtctggtttgccgcgccacccatttgaacaatttgataaccgtcatttaatactgagtattctgcggcgccattcatttcaatgtactgataccctccgttaatttcagtattattacttacaccaaattcttttatatgctgttctccgccaggattgattttcgtattattggcggtcccataaaccagctggatatctttttccaggacaacaccatcaactgtctcaccatactcaacagttgatgccaatgctatgggtgaatataaggaaggtgcgaataagcggggaaattcttctcggctgactcagtcatttcatttcttcatgtttgagccgattttttctcccgtaaatgccttgaatcagcctatttagaccgtttcttcgccatttaaggcgttatccccagtttttagtgagatctctcccactgacgtatcatttggtccgcccgaaacaggttggccagcgtgaataacatcgccagttggttatcgtttttcagcaaccccttgtatctggctttcacgaagccgaactgtcgcttgatgatgcgaaatgggtgctccaccctggcccggatgctggctttcatgtattcgatgttgatggccgttttgttcttgcgtggatgctgtttcaaggttcttaccttgccggggcgctcggcgatcagccagtccacatccacctcggccagctcctcgcgctgtggcgccccttggtagccggcatcggctgagacaaattgctcctctccatgcagcagattacccagctgattgaggtcatgctcgttggccgcggtggtgaccaggctgtgggtcaggccactcttggcatcgacaccaatgtgggccttcatgccaaagtgccactgattgcctttcttggtctgatgcatctccggatcgcgttgctgctctttgttcttggtcgagctgggtgcctcaatgatggtggcatcgaccaaggtgccttgagtcatcatgacgcctgcttcggccagccagcgattgatggtcttgaacaattggcgggccagttgatgctgctccagcaggtggcggaaattcatgatggtggtgcggtccggcaaggcgctatccagggataaccgggcaaacagacgcatggaggcgatttcgtacagagcatcttccatcgcgccatcgctcaggttgtaccaatgctgcatgcagtgaatgcgtagcatggtttccagcggataaggtcgccggccattaccagccttggggtaaaacggctcgatgacttccaccatgttttgccatggcagaatctgctccatgcgggacaagaaaatctcttttctggtctgacggcgcttactgctgaattcactgtcggcgaaggtaagttgatgactcatgatgaaccctgttctatggctccagatgacaaacatgatctcatatcagggacttgttcgcaccttccataacgctgtagccaccagaacagatattgcggaacgacaaagagaaacagaaccagattgatgcattgagctttcatcctatgaaattaattgctgttaaaagcattgggtacagaaaatacccatagctccatacccggagtcagtttttaaaaactgtttaaagaaatgcacaagtattgtgattgattttttagttgtttttcttgatgagaagctgatgcaaaattccgtctttataatgaaaatgatgccaaagcgaacgacaaggttgtagttttcactacatgtccatacataaaatggggtaacattcacgcgcctggtagcgttaccaacgctacgctcaaacataatgattctaataaaacctcaggagactactatgcctgaagcaacaccttttcaggtgatgattgtggatgatcatccacttatgcgacgcggtgttcgtcagttactggagcttgatcctggctctgaagtggtcgccgaagcgggcgacggcgcgagcgctatcgatctggcgaatagactggatatcgacgtgatcttgctggatctcaatatgaaaggtatgagtggcctggatactctcaatgccttgcgcagggatggcgttaccgcgcaaattattatcctgaccgtatccgatgcctccagcgatgtctttgcgctgatagacgcaggcgcagacggttatctgttgaaagacagcgacccggaagtattgctggaagcgattcgtgccggagcgaaaggcagcaaagtctttagcgaacgcgtcaatcagtacttacgtgaacgtgaaatgtttggcgcggaagaagatcccttcagcgtgctgacggagcgcgagctggatgttctgcacgagctggcacaggggctgtcaaataaacagattgcctcggtgttgaatatttccgagcagacagtaaaagtacatattcgcaatctgctgcgtaaactcaatgtccgctcacgcgtggcggccaccattctgttcctgcaacaacgcggggcacaataaaaatagcccgatggatttatcatcgggctgagatttatgacaaacgcaaaactgcctgatgcgctacgcttatcaggcctacgtggatcgatcaatttattgaatttacacaattttgtaggccggataaggcgttcacgccgcatccggcataaactaagcgcactttgtcaacagtctagcccgatggcatcaccatcgggcctctttttatttactctcctgcggcgacaaatgttgcatcgcctgcgcgatactacgttcaatcaccgcacggcgagtatcgttggcaggtaagagtttcaacatcatctcccacgcggcaacggcttcgccaaatcgctgctgctcaaaggcattaaacgcatacatgcttagcacacggatattgctatggtccgttctcaccagctgacgtagcagttcaccgccgaggcggttgtcgttgggatcagatgaacgagtcaacgcttcagcgtatcccag

**f_6_/r_6_ (*E. coli*^2.28/2.36^)：3127 bp**

gatctgctcgacgttcgcgcattactggagggcgaatcggcaaggctggcggcaacgctgggaacgcaggctgattttgttgtgataacccgctgttatgaaaaaatgctcgccgccagtgagaacaacaaagagatttcgctgatcgaacatgcgcagttggatcacgctttccatctcgccatttgtcaggcttctcacaatcaggtgctggtgtttacgctgcaatcattgaccgatctgatgtttaattcagtgtttgccagcgtaaataatctctaccatcgaccacagcaaaaaaagcagatcgatcgccagcatgcgcggatctacaacgcggtgttgcagcggctgccgcacgtcgcccagcgcgcagcacgcgatcatgtgcggaccgtgaaaaagaatctccacgatatcgagctggaaggccaccatttgattcgctcggcggtgccgctggagatgaacctgagttagctggtattaaatctgcttttcatacaatcggtaacgcttgtacggctccgccccaatgcgttccagcatgttattcatgcctgtattggtttcgaggatccatgacatctccagcgcatcgatcttccggcgggcaaacggatcgcgtaaggcttcaatcaataacagcgcaatcaccgggccgatgcggctgaactgatactcgtcgcgcacgcccatcagcggtactcgcgcagttcgcacaccgctgactttcaaacgccacagcaattttgcccagccgaagggaaagagcgatccgttcagatcggcaatcgcctcgttgatgttcggcaagccgacaataaacgcgcagggtgcagaatcaatctcagcgatatagatcatatcgtccggcaccagatatttaagttgatcgcccatggtcgcgaattcatgttcggtaaacggcacaaatccccagttgtgctgccagccagagttgaaaatctcacgcaggatctgcatctcttcggcaaaccgctgacgattgatgcagcgaatggtcacctttttgcgcacctgatccatcagtttttttagcgccggagagaaagtgagatcggttcgctgcatccaccacgccagtaaatcaatgcctttgtgataacccagttgttcaatatgcgcggcataccacggtttgccgtgtggcatcatcgcacagggtggtgtgtcaaaaccttcaatcagtaatccgctttcctgattgatattcaggctgaaaggaccgctgatcttacttgcaccttgtgacttcaaccacgcttccgctgcgccaaacaacgcggcaaaaacctgcggatcatcaatggcgtcaatcatgccgaaatgaccggtatctttgccgtaacgctcgcggtgcaaggtatctatttgcgcggtaatacgcccaactatctgccccgctttttttgccacccacgcctgccagatgatatggtccgtccccggatttttcgcagacaaatgctcgttgcgttcaatgaataaagggggtatccagtttggatcgtcgggataaagtgatgacggaaaagcgataaatgccttaaggtcatttttattaaggaaggtgcgaacaagtccctgatatgagatcatgtttgtcatctggagccatagaacagggttcatcatgagtcatcaacttaccttcgccgacagtgaattcagcagtaagcgccgtcagaccagaaaagagattttcttgtcccgcatggagcagattctgccatggcaaaacatggtggaagtcatcgagccgttttaccccaaggctggtaatggccggcgaccttatccgctggaaaccatgctacgcattcactgcatgcagcattggtacaacctgagcgatggcgcgatggaagatgctctgtacgaaatcgcctccatgcgtctgtttgcccggttatccctggatagcgccttgccggaccgcaccaccatcatgaatttccgccacctgctggagcagcatcaactggcccgccaattgttcaagaccatcaatcgctggctggccgaagcaggcgtcatgatgactcaaggcaccttggtcgatgccaccatcattgaggcacccagctcgaccaagaacaaagagcagcaacgcgatccggagatgcatcagaccaagaaaggcaatcagtggcactttggcatgaaggcccacattggtgtcgatgccaagagtggcctgacccacagcctggtcaccaccgcggccaacgagcatgacctcaatcagctgggtaatctgctgcatggagaggagcaatttgtctcagccgatgccggctaccaaggggcgccacagcgcgaggagctggccgaggtggatgtggactggctgatcgccgagcgccccggcaaggtaagaaccttgaaacagcatccacgcaagaacaaaacggccatcaacatcgaatacatgaaagccagcatccgggccagggtggagcacccatttcgcatcatcaagcgacagttcggcttcgtgaaagccagatacaaggggttgctgaaaaacgataaccaactggcgatgttattcacgctggccaacctgtttcgggcggaccaaatgatacgtcagtgggagagatctcactaaaaactggggataacgccttaaatggcgaagaaacggtctaaataggctgattcaaggcatttacgggagaaaaaatcggctcaaacatgaagaaatgaaatgactgagtcagccgagaagaatttccccgcttattcgcaccttccctaaatcaggtcatacgcttcgagatacttaacgccaaacaccagcgaaatgagcggcttgccgacgatcaacaccgcgagcgccaccagaataccgattccgcccgccagtaaaccggacttcacacctaacaaccacggtctggtggtgcgcggatctaaacgcatcacctctgggtaaaaacttttacccagcaaacccgctggcgttccggcggcgtcgaagaatgtcatggcgattttaaataacccggcggcagcgggtcctaacacgatccccaccaacactgtgctgcacgagttacgcgccgaccagatggagtgggcaatgttggttgaccag

**f_5_/f_6_ (*E. coli*^Mut^)： 3094 bp**

ctgcgccataaacctgtagcacaccattattgagcgtagtctggtttgccgcgccacccatttgaacaatttgataaccgtcatttaatactgagtattctgcggcgccattcatttcaatgtactgataccctccgttaatttcagtattattacttacaccaaattcttttatatgctgttctccgccaggattgattttcgtattattggcggtcccataaaccagctggatatctttttccaggacaacaccatcaactgtctcaccatactcaacagttgatgccaatgctatgggtgaatataaggaaggtgcgaataagcggggaaattcttctcggctgactcagtcatttcatttcttcatgtttgagccgattttttctcccgtaaatgccttgaatcagcctatttagaccgtttcttcgccatttaaggcgttatccccagtttttagtgagatctctcccactgacgtatcatttggtccgcccgaaacaggttggccagcgtgaataacatcgccagttggttatcgtttttcagcaaccccttgtatctggctttcacgaagccgaactgtcgcttgatgatgcgaaatgggtgctccaccctggcccggatgctggctttcatgtattcgatgttgatggccgttttgttcttgcgtggatgctgtttcaaggttcttaccttgccggggcgctcggcgatcagccagtccacatccacctcggccagctcctcgcgctgtggcgccccttggtagccggcatcggctgagacaaattgctcctctccatgcagcagattacccagctgattgaggtcatgctcgttggccgcggtggtgaccaggctgtgggtcaggccactcttggcatcgacaccaatgtgggccttcatgccaaagtgccactgattgcctttcttggtctgatgcatctccggatcgcgttgctgctctttgttcttggtcgagctgggtgcctcaatgatggtggcatcgaccaaggtgccttgagtcatcatgacgcctgcttcggccagccagcgattgatggtcttgaacaattggcgggccagttgatgctgctccagcaggtggcggaaattcatgatggtggtgcggtccggcaaggcgctatccagggataaccgggcaaacagacgcatggaggcgatttcgtacagagcatcttccatcgcgccatcgctcaggttgtaccaatgctgcatgcagtgaatgcgtagcatggtttccagcggataaggtcgccggccattaccagccttggggtaaaacggctcgatgacttccaccatgttttgccatggcagaatctgctccatgcgggacaagaaaatctcttttctggtctgacggcgcttactgctgaattcactgtcggcgaaggtaagttgatgactcatgatgaaccctgttctatggctccagatgacaaacatgatctcatatcagggacttgttcgcaccttccttaataaaaatgaccttaaggcatttatcgcttttccgtcatcactttatcccgacgatccaaactggataccccctttattcattgaacgcaacgagcatttgtctgcgaaaaatccggggacggaccatatcatctggcaggcgtgggtggcaaaaaaagcggggcagatagttgggcgtattaccgcgcaaatagataccttgcaccgcgagcgttacggcaaagataccggtcatttcggcatgattgacgccattgatgatccgcaggtttttgccgcgttgtttggcgcagcggaagcgtggttgaagtcacaaggtgcaagtaagatcagcggtcctttcagcctgaatatcaatcaggaaagcggattactgattgaaggttttgacacaccaccctgtgcgatgatgccacacggcaaaccgtggtatgccgcgcatattgaacaactgggttatcacaaaggcattgatttactggcgtggtggatgcagcgaaccgatctcactttctctccggcgctaaaaaaactgatggatcaggtgcgcaaaaaggtgaccattcgctgcatcaatcgtcagcggtttgccgaagagatgcagatcctgcgtgagattttcaactctggctggcagcacaactggggatttgtgccgtttaccgaacatgaattcgcgaccatgggcgatcaacttaaatatctggtgccggacgatatgatctatatcgctgagattgattctgcaccctgcgcgtttattgtcggcttgccgaacatcaacgaggcgattgccgatctgaacggatcgctctttcccttcggctgggcaaaattgctgtggcgtttgaaagtcagcggtgtgcgaactgcgcgagtaccgctgatgggcgtgcgcgacgagtatcagttcagccgcatcggcccggtgattgcgctgttattgattgaagccttacgcgatccgtttgcccgccggaagatcgatgcgctggagatgtcatggatcctcgaaaccaatacaggcatgaataacatgctggaacgcattggggcggagccgtacaagcgttaccgattgtatgaaaagcagatttaataccagctaactcaggttcatctccagcggcaccgccgagcgaatcaaatggtggccttccagctcgatatcgtggagattctttttcacggtccgcacatgatcgcgtgctgcgcgctgggcgacgtgcggcagccgctgcaacaccgcgttgtagatccgcgcatgctggcgatcgatctgctttttttgctgtggtcgatggtagagattatttacgctggcaaacactgaattaaacatcagatcggtcaatgattgcagcgtaaacaccagcacctgattgtgagaagcctgacaaatggcgagatggaaagcgtgatccaactgcgcatgttcgatcagcgaaatctctttgttgttctcactggcggcgagcattttttcataacagcgggttatcacaacaaaatcagcctgcgttcccagcgttgccgccagccttgccgattcgccctccagtaatgcgcgaacgtcgagcagatc

**r_5_/r_6_ (*E. coli*^Mut^)：3058 bp**

ctggtcaaccaacattgcccactccatctggtcggcgcgtaactcgtgcagcacagtgttggtggggatcgtgttaggacccgctgccgccgggttatttaaaatcgccatgacattcttcgacgccgccggaacgccagcgggtttgctgggtaaaagtttttacccagaggtgatgcgtttagatccgcgcaccaccagaccgtggttgttaggtgtgaagtccggtttactggcgggcggaatcggtattctggtggcgctcgcggtgttgatcgtcggcaagccgctcatttcgctggtgtttggcgttaagtatctcgaagcgtatgacctgatttagggaaggtgcgaataagcggggaaattcttctcggctgactcagtcatttcatttcttcatgtttgagccgattttttctcccgtaaatgccttgaatcagcctatttagaccgtttcttcgccatttaaggcgttatccccagtttttagtgagatctctcccactgacgtatcatttggtccgcccgaaacaggttggccagcgtgaataacatcgccagttggttatcgtttttcagcaaccccttgtatctggctttcacgaagccgaactgtcgcttgatgatgcgaaatgggtgctccaccctggcccggatgctggctttcatgtattcgatgttgatggccgttttgttcttgcgtggatgctgtttcaaggttcttaccttgccggggcgctcggcgatcagccagtccacatccacctcggccagctcctcgcgctgtggcgccccttggtagccggcatcggctgagacaaattgctcctctccatgcagcagattacccagctgattgaggtcatgctcgttggccgcggtggtgaccaggctgtgggtcaggccactcttggcatcgacaccaatgtgggccttcatgccaaagtgccactgattgcctttcttggtctgatgcatctccggatcgcgttgctgctctttgttcttggtcgagctgggtgcctcaatgatggtggcatcgaccaaggtgccttgagtcatcatgacgcctgcttcggccagccagcgattgatggtcttgaacaattggcgggccagttgatgctgctccagcaggtggcggaaattcatgatggtggtgcggtccggcaaggcgctatccagggataaccgggcaaacagacgcatggaggcgatttcgtacagagcatcttccatcgcgccatcgctcaggttgtaccaatgctgcatgcagtgaatgcgtagcatggtttccagcggataaggtcgccggccattaccagccttggggtaaaacggctcgatgacttccaccatgttttgccatggcagaatctgctccatgcgggacaagaaaatctcttttctggtctgacggcgcttactgctgaattcactgtcggcgaaggtaagttgatgactcatgatgaaccctgttctatggctccagatgacaaacatgatctcatatcagggacttgttcgcaccttccataacgctgtagccaccagaacagatattgcggaacgacaaagagaaacagaaccagattgatgcattgagctttcatcctatgaaattaattgctgttaaaagcattgggtacagaaaatacccatagctccatacccggagtcagtttttaaaaactgtttaaagaaatgcacaagtattgtgattgattttttagttgtttttcttgatgagaagctgatgcaaaattccgtctttataatgaaaatgatgccaaagcgaacgacaaggttgtagttttcactacatgtccatacataaaatggggtaacattcacgcgcctggtagcgttaccaacgctacgctcaaacataatgattctaataaaacctcaggagactactatgcctgaagcaacaccttttcaggtgatgattgtggatgatcatccacttatgcgacgcggtgttcgtcagttactggagcttgatcctggctctgaagtggtcgccgaagcgggcgacggcgcgagcgctatcgatctggcgaatagactggatatcgacgtgatcttgctggatctcaatatgaaaggtatgagtggcctggatactctcaatgccttgcgcagggatggcgttaccgcgcaaattattatcctgaccgtatccgatgcctccagcgatgtctttgcgctgatagacgcaggcgcagacggttatctgttgaaagacagcgacccggaagtattgctggaagcgattcgtgccggagcgaaaggcagcaaagtctttagcgaacgcgtcaatcagtacttacgtgaacgtgaaatgtttggcgcggaagaagatcccttcagcgtgctgacggagcgcgagctggatgttctgcacgagctggcacaggggctgtcaaataaacagattgcctcggtgttgaatatttccgagcagacagtaaaagtacatattcgcaatctgctgcgtaaactcaatgtccgctcacgcgtggcggccaccattctgttcctgcaacaacgcggggcacaataaaaatagcccgatggatttatcatcgggctgagatttatgacaaacgcaaaactgcctgatgcgctacgcttatcaggcctacgtggatcgatcaatttattgaatttacacaattttgtaggccggataaggcgttcacgccgcatccggcataaactaagcgcactttgtcaacagtctagcccgatggcatcaccatcgggcctctttttatttactctcctgcggcgacaaatgttgcatcgcctgcgcgatactacgttcaatcaccgcacggcgagtatcgttggcaggtaagagtttcaacatcatctcccacgcggcaacggcttcgccaaatcgctgctgctcaaaggcattaaacgcatacatgcttagcacacggatattgctatggtccgttctcaccagctgacgtagcagttcaccgccgaggcggttgtcgttgggatcagatgaacgagtcaacgcttcagcgtatcccag

**Figures**


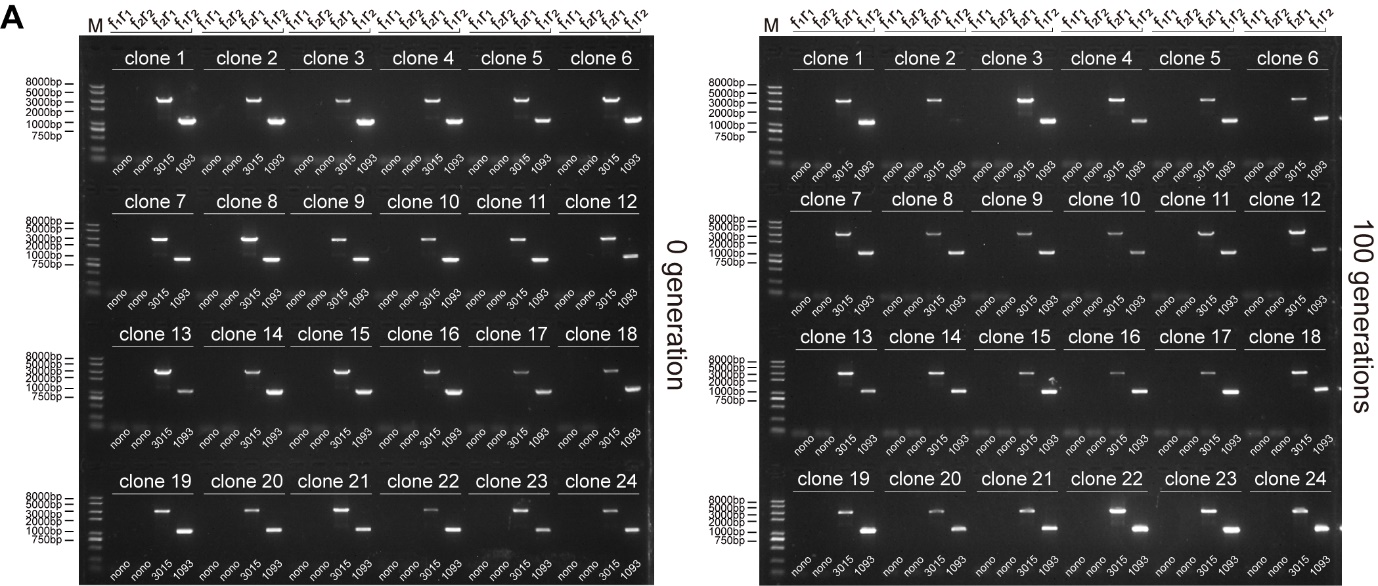

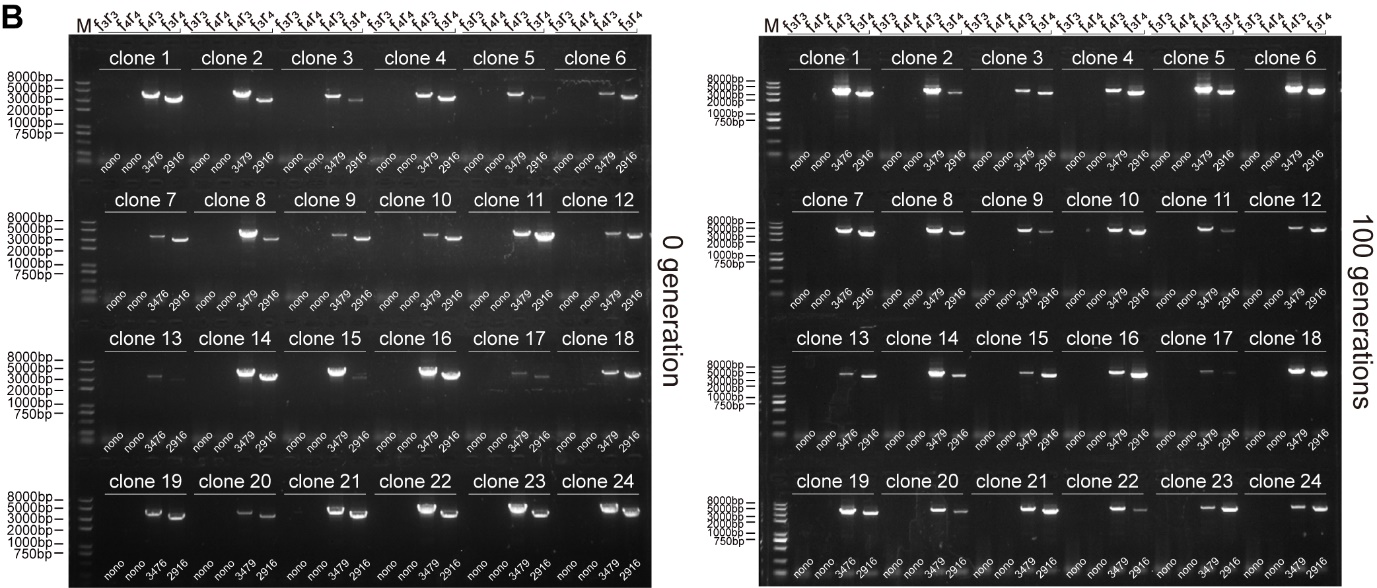


**Figure S1.** Stability of the chromosomal organization of *E. coli*^0.10/4.54^ and *E. coli*^2.28/2.36^. (A) Colony PCR analysis of the *E. coli*^0.10/4.54^ cells after culturing for more than 100 generations using the primer pairs f_1_/r_1_, f_2_/r_2_, f_1_/r_2_ and f_2_/r_1_. (B) Colony PCR analysis of *E. coli*^2.28/2.36^ after culturing for more than 100 generations using the primer pairs f_3_/r_3_, f_4_/r_4_, f_3_/r_4_ and f_4_/r_3_.


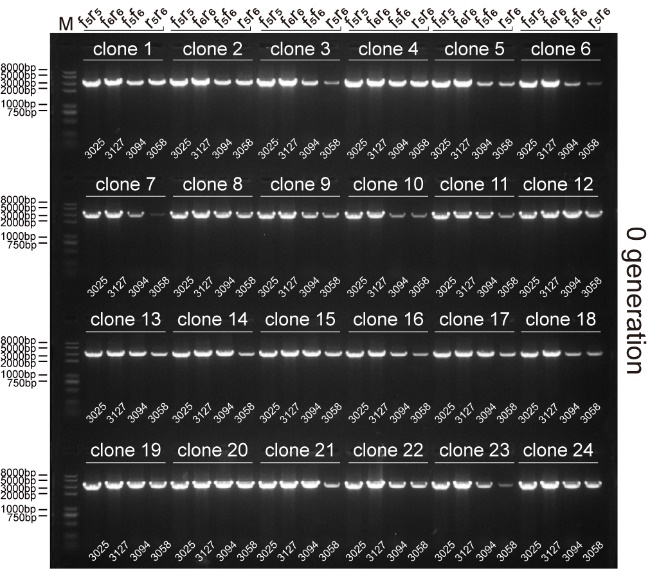

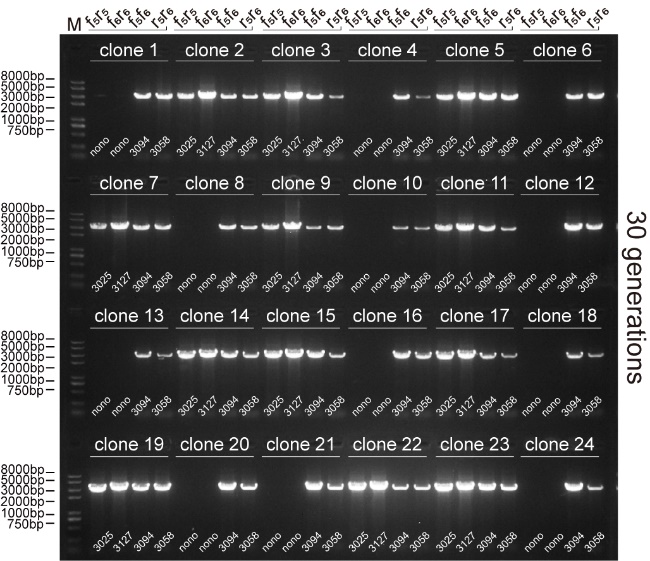

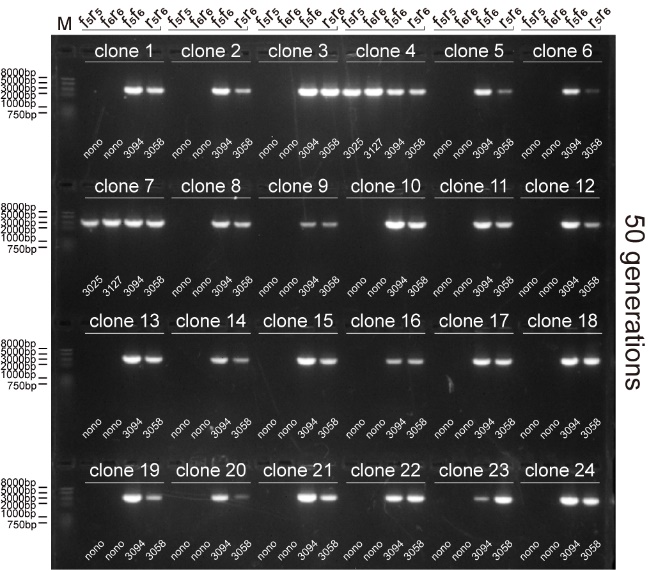

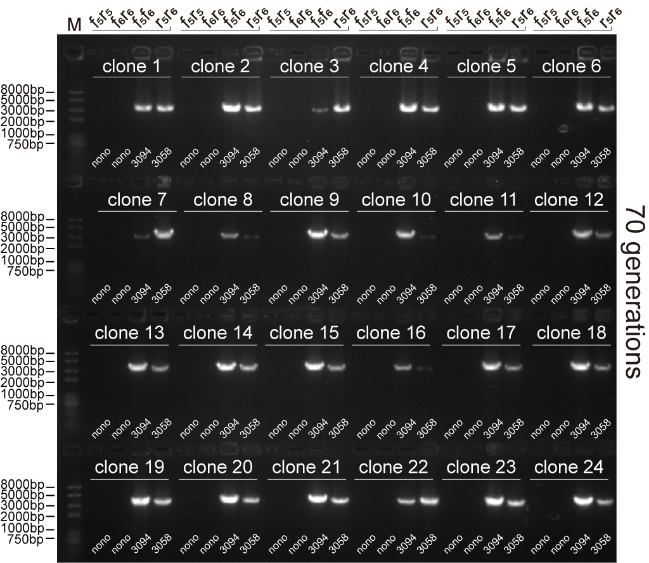

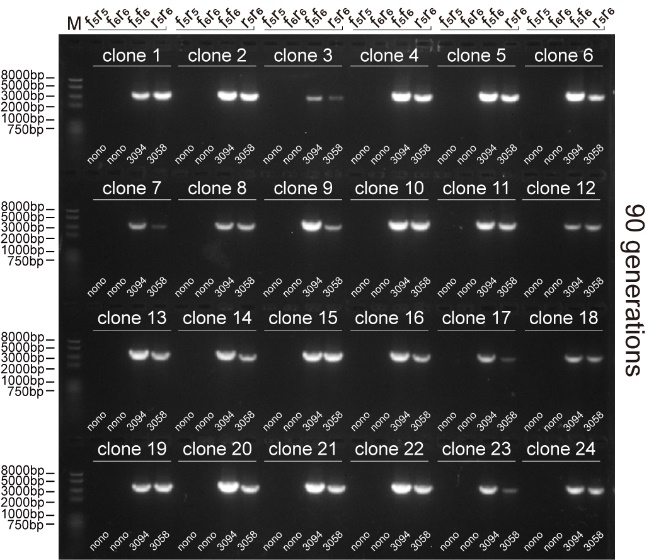

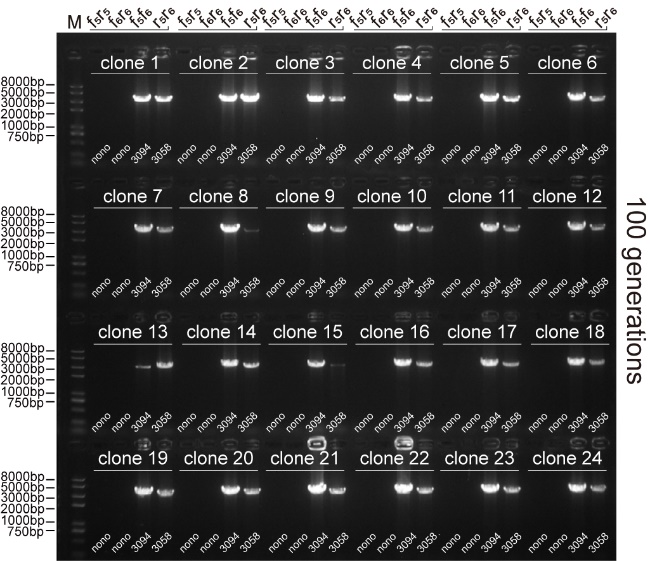


**Figure S2.** Stability of the chromosomal organization of *E. coli*^2.28/2.36^ (mix with *E. coli*^Mut^). A total of 11 rounds of inoculation were conducted, corresponding to approximately 100 generations. The culture of the first (0 generation), third (about 30 generations), fifth (about 50 generations), seventh (about 70 generations), ninth (about 90 generations) and last (about 100 generations) round of *E. coli*^2.28/2.36^ (mix with *E. coli*^Mut^) was spread on LB agar plates, 24 single colonies of each strain were selected and four pairs of primers f_5_/r_5_, f_6_/r_6_, f_5_/f_6_ and r_5_/r_6_ were used to investigate the speed of transition of genomic instability with the passage of generations.
